# Traction force and mechanosensitivity mediate species-specific implantation patterns in human and mouse embryos

**DOI:** 10.1126/sciadv.adr5199

**Published:** 2025-08-15

**Authors:** Amélie Luise Godeau, Anna Seriola, Oren Tchaicheeyan, Marc Casals, Denitza Denkova, Ester Aroca, Ot Massafret, Albert Parra, Maria Demestre, Anna Ferrer-Vaquer, Shahar Goren, Anna Veiga, Miquel Solé, Montse Boada, Jordi Comelles, Elena Martínez, Julien Colombelli, Ayelet Lesman, Samuel Ojosnegros

**Affiliations:** ^1^ Institute for Bioengineering of Catalonia (IBEC), The Barcelona Institute of Science and Technology (BIST), 08028 Barcelona, Spain.; ^2^ School of Mechanical Engineering, Faculty of Engineering, Tel Aviv University, Tel-Aviv, 55 Chaim Levanon St., Ramat Aviv 69978, Israel.; ^3^ Barcelona Stem Cell Bank, IDIBELL Programme for Regenerative Medicine, 08908, L’Hospitalet de Llobregat, Spain.; ^4^ Reproductive Medicine Unit, Department of Obstetrics, Gynaecology and Reproduction, Dexeus Mujer, Hospital Universitari Dexeus, Gran Vía Carles III, 71-75, 08028, Barcelona, Spain.; ^5^ Department of Electronics and Biomedical Engineering, University of Barcelona (UB), c/Martí i Franquès 1-11, 08028 Barcelona, Spain.; ^6^ Centro de Investigación Biomédica en Red (CIBER), Av. Monforte de Lemos 3-5, Pabellón 11, Planta 0, 28029 Madrid, Spain.; ^7^ Institute for Research in Biomedicine (IRB Barcelona), The Barcelona Institute of Science and Technology, Barcelona 08028, Spain.; ^8^ Center for Physics and Chemistry of Living Systems, Tel Aviv University, Tel-Aviv, 55 Chaim Levanon St., Ramat Aviv 69978, Israel.

## Abstract

The invasion of human embryos in the uterus overcoming the maternal tissue barrier is a crucial step in embryo implantation and subsequent development. Although tissue invasion is fundamentally a mechanical process, most studies have focused on the biochemical and genetic aspects of implantation. Here, we fill the gap by using a deformable ex vivo platform to visualize traction during human embryo implantation. We demonstrate that embryos apply forces remodeling the matrix with species-specific displacement amplitudes and distinct radial patterns: principal displacement directions for mouse embryos, expanding on the surface while human embryos insert in the matrix generating multiple traction foci. Implantation-impaired human embryos showed reduced displacement, as well as mouse embryos with inhibited integrin-mediated force transmission. External mechanical cues induced a mechanosensitive response, human embryos recruited myosin, and directed cell protrusions, while mouse embryos oriented their implantation or body axis toward the external cue. These findings underscore the role of mechanical forces in driving species-specific invasion patterns during embryo implantation.

## INTRODUCTION

Mammalian embryos initiate their development autonomously in the lumen of the mother’s uterus (*1*) before attaching to the endometrium, the tissue lining the uterus, and progressively establishing the interface with the mother. These events, termed implantation, have resulted from an evolutionary novelty of mammals and are at the origin of their prolonged intrauterine gestation (*2*). Implantation failure is one of the main causes of infertility, accounting for 60% of miscarriages (*3*, *4*). During implantation, the epiblast of the blastocyst differentiates to eventually give rise to all the embryonic tissues (*5*) by transiting from a spherical structure which already has a symmetry axis (polar and apolar) to a postimplantation bilateral symmetry with defined anterior-posterior (A-P) and proximal-distal (P-D) body axes (*6*).

The blastocyst invasion is mediated by its outer layer, the trophectoderm (TE), that penetrates the endometrium upon attachment, connecting the embryo with the maternal blood supply (*7*–*9*) and progressively giving rise to the placenta. The depth of the trophoblast invasion differs across all placental mammals (*2*, *10*). Human embryos grow interstitially, fully encapsulating themselves into the uterine tissues (*11*), whereas mouse embryos invade more superficially but become engulfed into a uterine crypt (*12*, *13*). In both species, the embryo secretes metalloproteinases to degrade the collagen-rich (*14*–*16*) extracellular matrix (ECM) and facilitate the invasion (*17*, *18*). However, matrix degradation alone cannot account for implantation since the embryo still needs to exert forces to penetrate the fibrous stromal tissue that form the underlying layers of the uterus. However, so far, the relative inaccessibility of the process, especially in humans, has precluded recording embryo implantation in vivo, and, therefore, the available evidence about the mechanisms underlying implantation and invasion is only indirect, relying on imaging of fixed samples (*19*).

The role of mechanics during embryo morphogenesis is well established. Several studies indicate that contractility and mechanical forces influence pre- and postimplantation stages of mouse embryo development, such as compaction (*20*–*22*), lineage segregation (*23*, *24*), blastocoel (*25*) and egg cup formation (*26*, *27*), and A-P axis establishment (*28*, *29*). In addition, embryos experience external forces, e.g., uterine contractions (*30*, *31*), and apply forces to facilitate hatching (*32*). A recent study reported that 68% of lethal and subviable genetic mouse knockout lines show placental defects (*33*), highlighting that the trophoblast contribution to the development of the conceptus is a blind spot in embryo development. In human embryos, it has been suggested that the polar TE exerts a stretching force to mold the embryo into a disc shape (*34*). However, since most of these studies have focused on the epiblast, many unknowns remain on how mechanical forces affect the trophoblast and facilitate implantation.

To overcome the limitation associated with observing embryo implantation in vivo, we have developed an ex vivo implantation platform amenable to live fluorescence imaging and traction force microscopy. The system captures the dynamics of blastocyst implantation in four dimensions (4D) (*x*, *y*, *z*, and *t*) and peri-implantation development during the initial trophoblast invasion. By measuring the direct impact of the embryo on the matrix scaffold, we reveal the underlying mechanics of embryo implantation. We found that mouse and human embryos generated forces during implantation using a species-specific pattern. Moreover, we demonstrate that embryos are mechanosensitive and show how external forces could determine the orientation of the P-D axis in mouse and promote invasion in human embryos.

## RESULTS

### An ex vivo platform to study human and mouse embryo implantation

**Fig. 1.**
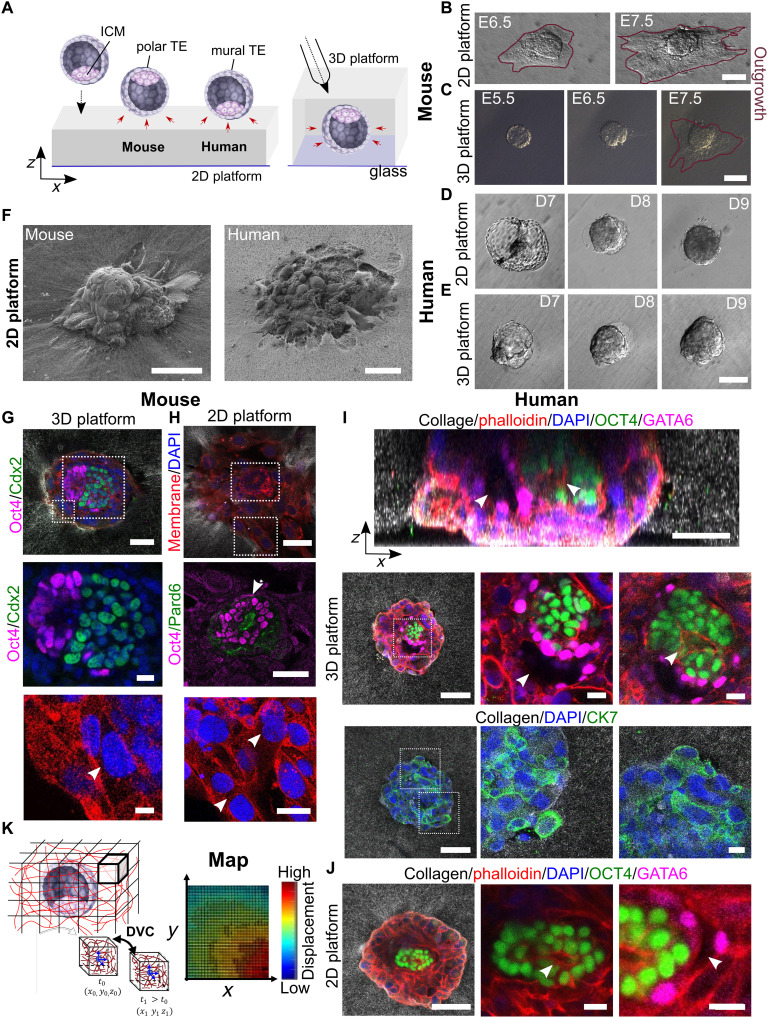
An ex vivo platform to study human and mouse embryo implantation. (**A**) Schematic illustration of mouse and human blastocyst implanting in the 2D and 3D platforms. Mouse embryos adhere on the mural and human blastocysts on the polar TE. (**B** to **E**) Snapshots of mouse or human embryo implanting on the 2D and 3D platforms. The outgrowth increased over time (red outline). Scale bars, 100 μm. (**F**) Scanning electron microscopy images of (left) mouse and (right) human embryo implanted on the 2D platform. Scale bars, 50 μm. (**G**) 4′,6-Diamidino-2-phenylindole (DAPI), Oct4, and Cdx2 immunostaining of a mouse embryo expressing membrane-bound tdTomato implanted in the 3D platform at E7.5. Scale bars, 50 μm. The insets show epiblast, ExE, and trophoblast multinucleated cells (white arrowhead). Scale bar, 10 μm. (**H**) DAPI, Oct4, and Pard6 immunostaining of a mouse embryo expressing membrane-bound tdTomato implanted on the 2D platform. The insets show proamniotic cavity and multinucleated cells (white arrows). Scale bars, 100 and 50 μm (inset). (**I** and **J**) DAPI, phalloidin, OCT4, GATA6, and CK7 immunostaining of human embryos (representative of five embryos) at D10 implanted in the (I) 3D (top) orthogonal and (bottom) top view and (J) 2D platform. The white arrows point at the prospective yolk sac and proamniotic cavity. Scale bars, 100 μm, 50 μm (orthogonal view), and 20 μm (insets). (**K**) Schematic description of the digital volume correlation (DVC) analysis. Fiducial markers were tracked between time points, and a displacement map was generated. The amplitude of displacement is color-coded, and arrows indicate the direction of displacement.

Our ex vivo implantation platform includes type I collagen to mimic the ECM environment encountered by the preimplantation embryo in vivo (*14*) and globulin-rich protein supplement from human origin. To study the mechanical interaction of the embryo with its surroundings, we designed two complementary platform configurations. In the first one, blastocysts were let to settle on top of a 2D flat gel where, eventually, they bound to the collagen surface. In the second one, blastocysts were placed inside collagen drops close to the glass surface, where they become integrated in the matrix. The two setups allow to mimic the different states of early embryo implantation where the blastocyst encounters first a surface (2D platform) before either penetration of the stromal layer or by being engulfed by the surrounding tissue (3D platform) and thereafter transitioning to the peri-implantation embryo. These setups, hereafter referred to as 2D and 3D platforms, respectively (Fig. 1A), allowed us to study the impact of the different trophoblast cells, such as the polar, the mural, and the trophoblast giant cells (TGCs), on implantation. In addition, in the 3D platform, blastocysts are immobilized upon integration in the matrix thus embryo interdistance can be precisely controlled.

The mechanical characterization of the 2D platform using an atomic force microscope (AFM) reported a range of 10 to 20 kPa for the elastic modulus (fig. S1A), which was consistent with that of the mouse uterine environment (*29*). In human uteri, AFM measurements indicate a stiffness of 230 to 250 Pa for nonpregnant uterus and decidua parietalis and a stiffness of 1.2 kPa for decidua basalis (*35*). In the 2D platform, mouse blastocyst adhesion led to the formation of a trophoblast outgrowth, which expanded superficially on the collagen gel and increased over time (Fig. 1B and fig. S1B). In the 3D platform, mouse trophoblast outgrowth formed partially in the gel and on the glass surface, whereas the trophoblastic cells were free to adhere to the gel fibers in any direction (Fig. 1C). In contrast to mice, in the 2D platform, human embryos integrated into the collagen matrix, moderately increasing size and self-embedding instead of forming an outgrowth, ultimately being surrounded by collagen matrix as in the 3D platform (Fig. 1, D to F, and fig. S1B).

We studied the expression of lineage markers and cavity formation for the embryos of both species. The mouse embryo expressed Cdx2 and Oct4 (Fig. 1H), defining extraembryonic ectoderm (ExE) and epiblast, respectively, and continued developing until the egg cylinder stage (fig. S1, C and D). Pard6 (Fig. 1H), a marker of cell polarity, was located in the epiblast indicating the proamniotic cavity (Fig. 1H). In both platforms, the TE differentiated to TGCs, as shown by the formation of large multinucleated cells (Fig. 1, G and H). For human embryos, we verified embryonic development using morphological observations and staining for specific lineage markers including OCT4 for the inner cell mass (ICM) and the epiblast, GATA6 for primitive endoderm, and cytokeratin 7 (CK7) for trophoblast, all of which have been used previously as standard markers (Fig. 1, I and J, and fig. S1E) (*36*–*38*). OCT4-expressing cells at the center of the implanting embryo defined the perimeter of the proamniotic cavity. GATA6 expression set the boundary of a prospective yolk sac cavity (Fig. 1, I and J) in both 2D and 3D platforms (*36*). Trophoblast cells were positive for CK7 (Fig. 1I) confirming proper TE lineage. Overall, this suggests that the implantation platforms promote implantation while conserving expression of embryonic lineage markers.

A reporter mouse line expressing membrane-tethered tdTomato allowed the time-lapse visualization of the mouse blastocyst morphology in 3D at high resolution (movie S1). For human blastocysts, an autofluorescence signal resulting from multiphoton illumination at the near-infrared range was used to generate 3D reconstructions on time-lapse movies (movie S2). Reflection microscopy revealed the matrix fibers in label-free conditions and minimal illumination and photodamaging, thanks to the strong signal from scattered light. To capture the blastocyst-induced local deformation of the matrix, we used a digital volume correlation (DVC) algorithm where the gel fibers were used as fiducial markers for real-time tracking (Fig. 1K) (*39*). From the DVC, we derived displacement maps, showing the direction and amplitude of such displacements, thus revealing the areas where the embryo was applying force. Overall, our platforms allow us to study the dynamics of human and mouse embryo implantation.

### Anisotropic displacement patterns emerge during mouse embryo invasion

**Fig. 2.**
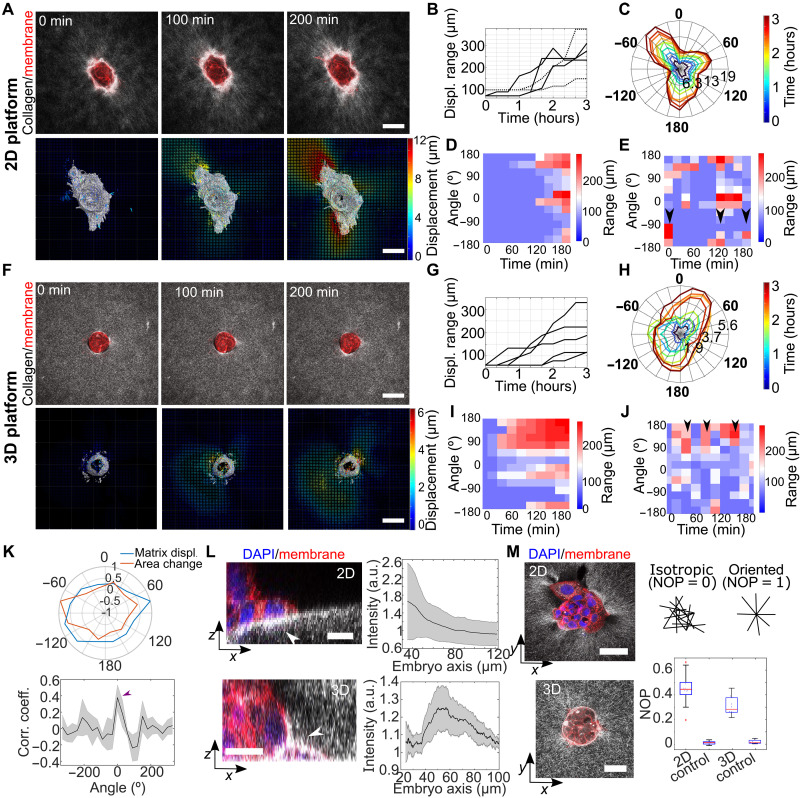
Anisotropic displacement patterns emerge during mouse embryo invasion. (**A** and **F**) Top: Time-lapse of embryos (membrane-bound tdTomato, red) deforming collagen matrix (light scattering) during implantation in 2D and 3D platforms (single *z* plane). Bottom: 3D maps of cumulative collagen displacement, color-coded by amplitude with displacement direction shown by arrows. Scale bars, 100 μm. (**B** and **G**) Plots of distance from embryo at which the cutoff displacement is observed over time. The displacement range increased over time. Cutoff: 1.2 μm (solid line) and 3.4 μm (dashed line), *N* = 2 and *n* = 5 [2D platform in (B)]. Cutoff: 1.9 μm, *N* = 2 and *n* = 5 [3D platform in (G)]. (**C** and **H**) Polar plot of isodisplacement (cumulative) contour lines generated by single embryos on the 2D or 3D platform, respectively. Time is color-coded. (**D** and **I**) Heatmap of radial cumulative (**E** and **J**) relative displacement for a representative embryo. Collagen displacement increased along two main axes, with displacement axes varying over time. Black arrows show recurrent displacement. (**K**) Top: Polar plot of collagen displacement (blue) and outgrowth (orange) for a representative embryo. Bottom: Average correlation, with a peak at 0 (purple arrow). Error: SEM. *N* = 2, *n* = 5 embryos. (**L**) Orthogonal snapshots of embryos (tdTomato, red) implanting in 2D (top left) and 3D (bottom left) platforms (DAPI, blue). White arrow, increased collagen signal. Scale bars, 20 μm. Right: Radial collagen intensity, peaking beneath embryos. *N*_2D_ = 11 embryos, *n*_3D_ = 8, *N* > 3. (**M**) Snapshots of embryos (tdTomato, red) implanting in 2D and 3D platforms (DAPI, blue). Scale bars, 20 μm (2D) and 50 μm (3D). Schematic of nematic order parameter (NOP: 0 = random, 1 = aligned). NOP distribution shows radial collagen organization in both platforms. *n* = 14, *N* > 3, *n* = 6; control: *N* = 2 z-stacks. a.u., arbitrary units.

Initial attachment of the mouse blastocyst was followed by trophoblast invasion and proliferation in both platforms. At this point, the embryo strongly applied pulling forces evident as a discernible movement of the fibrous matrix toward the embryo (Fig. 2A and movies S1 and S2).

The matrix displacement increased over time compared to the initial time point (cumulative displacement) and propagated in the collagen matrix in both the 2D and 3D platforms (Fig. 2, A to C and F to H, and fig. S2A). Long-range transmission of forces is facilitated in the matrix due to the nonlinear mechanical properties of collagen (*40*–*43*). The amplitude of the matrix displacement during the assay was similar for the 2D and 3D platforms (fig. S5B). However, the collagen displacement was not isotropic, with preferential axes of displacement emerging over time (Fig. 2, C to H). Individual embryos displaced collagen in two to three main directions in the 2D platform and on one to two in the 3D platform (Fig. 2, D to I). The preferential directions for the embryo outgrowth typically corresponded with the main displacement axes (Fig. 2K). This was consistent with a feedback mechanism in which the force applied to the collagen caused an accumulation of fibers and matrix stiffening (*44*), which could facilitate higher force generation and more spreading (*45*). The relative matrix displacement between two consecutive time points indicated that traction was not applied continuously but fluctuated in space and time. Displacement was not restricted to the preferential direction and was observed at any location around the embryo (Fig. 2, E to J, and fig. S2A), suggesting multiple anchoring points to the matrix. No uniform isotropic displacement was observed, neither for cumulative nor relative displacement.

We quantified the impact of blastocyst implantation on collagen distribution. A fiber densification and radial collagen alignment were observed for both platforms compared to the blastocyst-free matrix (Fig. 2, L and M, and fig. S2, B and C). In the 2D platform, the fiber densification decreased with distance to the embryo center, whereas, in the 3D platform, fiber densification led to a rim formation. These results indicate that the blastocysts remodeled the collagen and aligned the fibers around them during implantation, as reported for other systems, such as cancer spheroids (*46*). Flipping the plate upside down during polymerization (“hanging drop culture”) allowed us to rule out the possibility that the compression of fibers during polymerization acted as a factor for densification (fig. S2, D and E). The long-term hanging drop culture also dismissed purely gravitational effects in fiber densification (fig. S2, D to F).

In the 2D platform, the mouse blastocysts embedded slightly in the collagen matrix, and the applied traction led to an indentation of the collagen surface (fig. S3C). We investigated whether we could influence the implantation depth by varying the stiffness or matrix composition. Therefore, we diluted the collagen to 1.5 mg/ml leading to a stiffness of 188 Pa and let the embryos invade on this matrix (fig. S3, A and F). The outgrowth area decreased to 0.41 ± 0.22 mm^2^, and the depth slightly increased to 40.9 ± 10.5 μm (fig. S3, A, C, and E). Next, we used Matrigel for our 2D platform, a standard component for periembryo culture (*38*, *47*) and measured stiffness by AFM measurement 279 Pa (fig. S3F). When letting the embryos attach to the Matrigel platform, no spreading of the outgrowth on the surface could be observed leading to a much-reduced size of the embryos to 0.19 ± 0.05 mm^2^ compared to collagen control (fig. S3, A, D, and E). Adding collagen (e.g., 20%) to the Matrigel solution reinstated the outgrowth formation (fig. S3B). Nevertheless, the embryos were invading the Matrigel as they were deeply embedding into the gel reaching a depth of 90.2 ± 21.7 μm (fig. S3, C and E). The results suggest that the invasion behavior of the mouse embryo is coupled to the stiffness or composition or a combination of both properties of the substrate, although the individual contributions of each are difficult to disentangle. This hints at a competition between implantation depth and outgrowth size. Overall, mouse embryos pull and generate large matrix displacements which remodel collagen fibers in their vicinity, leading to increased collagen density and alignment.

### Matrix displacement pattern of human embryo invasion

**Fig. 3.**
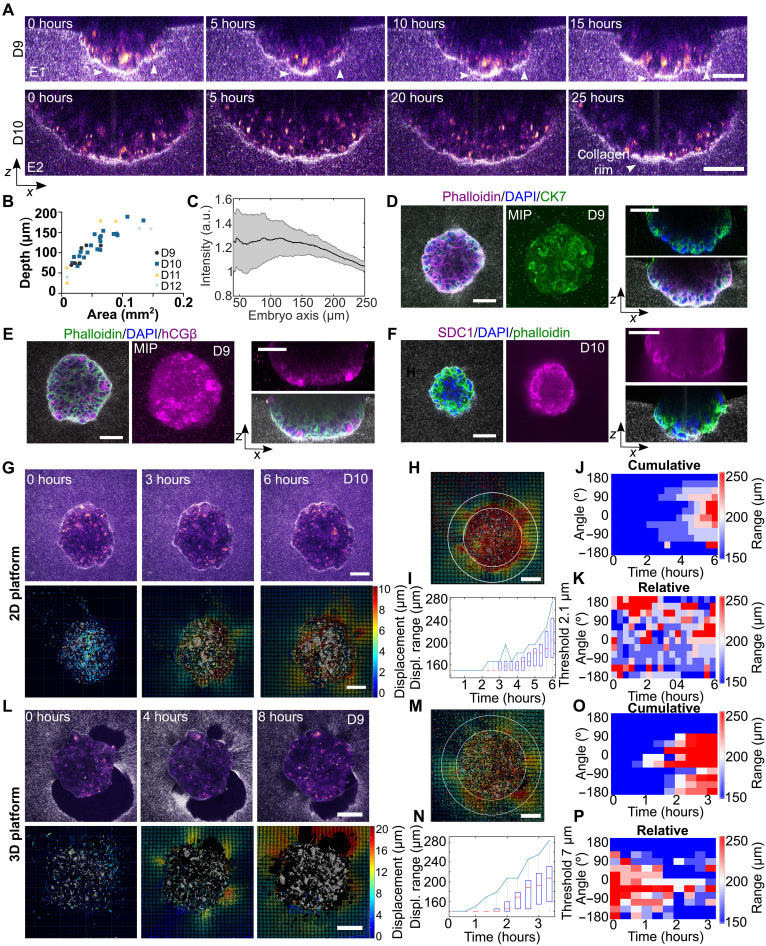
Matrix displacement pattern of human embryo invasion. (**A**) Orthogonal view of time-lapse images of two human embryos at (top) D9 and (bottom) D10, respectively, implanting in a 2D platform. The image of the matrix fibers and deformations was captured using light scattering, and the embryo was captured using autofluorescence and multiphoton illumination. Embryos integrate into the matrix (white arrows). (**B**) Implantation depth of human embryos on a 2D platform depending on size and time. *N* > 3, *n* = 36. (**C**) Plot of the average radial collagen intensity along the embryo axis, and the signal is maximal underneath the embryo. *N* = 8 embryos, *N* > 3. (**D** and **E**) Top and side views of immunostained human embryo implanted on a 2D platform with DAPI (blue) (D), phalloidin (purple), and CK7 (green). (E) Phalloidin (green) and hCGβ (purple). MIP, maximum intensity projection. (**F**) Phalloidin (green) and syndecan-1 (SDC1) (purple). Representative embryo of four to six. (**G** and **L**) Top: Time-lapse images (single *z* plane) of a human embryo implanting in the 2D or 3D platform. The image of the matrix fibers and deformations were captured using light scattering, and the embryo was captured using autofluorescence and multiphoton illumination. Bottom: Corresponding maps of cumulative collagen displacement of the entire 3D volume generated by single embryos. The displacement amplitude is color-coded, and the displacement direction is indicated with arrows. Scale bars, 100 μm. (**H** and **M**) Outline of the radius for calculated displacement range. (**I** and **N**) Graph showing the distance from the embryo at which 2.1- or 7.1-μm displacement is observed. The collagen displacement range increases over time around the embryo. (**J** and **O**) Heatmap of the radial distribution of cumulative and (**K** and **P**) relative displacement for a representative embryo on the 2D or 3D platform. Displacement is uniform around the embryo. Repetitive collagen displacement occurs around the embryo. Representative embryos for *n*_2D_ = *n*_3D_ = 4 embryos. Scale bars, 100 μm.

**Fig. 4.**
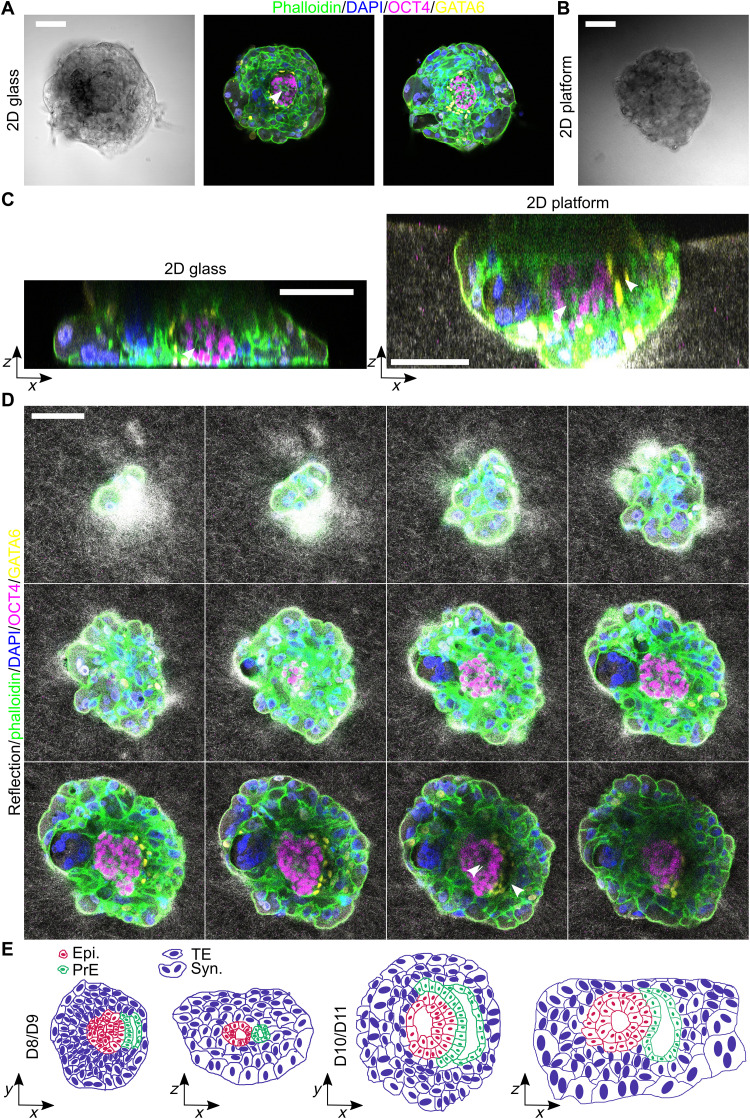
Human embryo adhesion to glass plate and implantation in 2D platform. (**A**) Immunostaining of a human embryo adhered to a 2D glass plate with phalloidin (green), OCT4 (purple), DAPI (blue), and GATA6 (yellow). The embryo shows OCT4-positive epiblast with proamniotic cavity. Large multinucleated cells appear at the rim of the embryo on a glass plate. (**B**) Snapshot of a bright-field image of a human embryo implanted in a 2D platform. (**C**) Side view of a human embryo implanted on (left) a glass surface and (right) a 2D platform with phalloidin (green), OCT4 (purple), DAPI (blue), and GATA6 (yellow). The embryo on the 2D platform shows a larger height compared to the embryo on the glass surface. (**D**) Series of confocal *z* sections. The embryo shows OCT4-positive epiblast with proamniotic cavity and prospective yolk sac cavity surrounded by GATA6-positive cells. Various cell layers of cytotrophoblast and syncytiotrophoblast are located between the epiblast and the matrix. (**E**) Schematic illustration of human peri-implantation development based on our results and the Carnegie series. The distribution of syncytiotrophoblast in 2D and 3D platforms differs from embryo culture on glass. Epiblast (Epi) in red, primitive endoderm (PrE) in green, TE, and syncytiotrophoblast (Syn) in blue. All embryos at D11, and all scale bars are 100 μm.

Human blastocysts attached with a rate of 72% on the 2D platform (fig. S4A) with the polar TE, located at the embryonic pole in contact with the ICM (fig. S4B). The attachment was followed by an invasion in the matrix where human blastocyst partially integrated into the gel representing a major difference to mouse implantation where only minimal embedding was observed (Fig. 3A and movie S3). Depth of integration and embryo size increased over time during invasion in the matrix (Fig. 3B) with the embryos reaching depth of up to 200 μm in the gel. Embryos within the matrix maintained a spherical shape, and no spreading on the surface occurred in contrast to embryos cultured on glass surfaces (Fig. 4, A and B).

Implantation in the 3D platform bypassed the attachment phase by embedding the embryo directly inside the matrix. We obtained 80% survival and invasion rate (fig. S4A), although embryo invasion was limited by the proximity of the glass surface. However, when the blastocysts were positioned with the ICM facing sideways, we observed a lateral translocation of the embryo into the matrix (fig. S4C and movie S4) leaving a void at their initial position. Regardless of the platform, the human blastocysts show motility in the matrix, in *z* direction in the 2D platform, and in *xy* in the 3D platform, illustrating the invasion capacity of human embryos.

The TE cells, which are the outer cells of the blastocyst in contact with the collagen matrix, show large nuclei and positive staining for the TE markers CK7 (Fig. 3D). In addition, at day 9 (D9) the TE cells start to express human chorionic gonadotropin β (hCGβ) and at D10 syndecan, both markers of syncytiotrophoblast (Fig. 3, E and F). Larger embryos show more hCGβ-positive cells (fig. S4D). At the same time, nascent lacunaes and multinucleated cells start to appear in the embryos of both 2D and 3D platforms (fig. S4G). Human chorionic gonadotropin was also detected in the media using a standard pregnancy test (fig. S4F) and by enzyme-linked immunosorbent assay (ELISA) with an average concentration of 109.5 ng/ml after 2 days of culture (fig. S4F), recapitulating hallmarks of syncytiotrophoblast differentiation (*48*). Concurrently, TE and syncytiotrophoblast precursor cells are located at the embryo matrix interface. During the course of invasion, multinucleated cells become larger with an increased number of nuclei per cell and more pronounced at the basal side of the embryo (fig. S4, E to G). At D12, cells infiltrate into the matrix detaching from the embryo mass with positive human leukocyte antigen-G (HLA-G) staining, a marker for extravillous trophoblast cells (fig. S4G). Together, the combination of molecular markers and cellular features suggests the differentiation of cells resembling syncytiotrophoblast in embryos cultured in our platforms.

Collagen accumulated underneath and around the embryo forming a rim, similar to the observation with mouse embryos but to a smaller extent (Figs. 2L and 3C). During the invasion, we observed large displacement toward the embryo center. The mechanical signature of the embryos on 2D and 3D platforms was similar as they were surrounded by collagen regardless of the platform setup. The displacement of the matrix increased over time and propagated in the collagen matrix (Fig. 3, H, I, M, and N). The DVC analysis showed a more regular spatial displacement during invasion suggesting multiple discrete anchoring points (Fig. 3, J to O, and movies S3 and S4), and no preferential axis of displacement as in mouse was identified. However, the distribution of displacement was not perfectly uniform (Fig. 3, J to O), and the relative DVC analysis revealed that pulling was not constant but varied over time and space, as also seen in mouse embryos, leading to fluctuating displacements (Fig. 3, K to P, and fig. S5A). This pulsatile behavior may serve the embryos to continuously sense the environment (*49*). Further, we found that the maximum displacement was higher for human embryos than for mouse embryos. Whereas mouse embryos showed a similar maximum deformation in both platforms, human embryos maximum deformations generated on a 3D platform were larger than on a 2D platform, possibly due to a larger volume of the embryo inside the matrix (fig. S5B). We tested the implantation behavior of low-quality embryos, as determined by their below-average size with the presence of dead cells. Low-quality embryos generated smaller displacement of the matrix compared to average sized embryos, with a limited displacement range, and did not show invasion into the matrix during the course of the time lapse (fig. S5, C and D). These human embryos also showed a reduced maximum displacement (fig. S5B); however, normalized by their size, they show a larger maximum displacement per contact area as well as normalized mouse embryos. These results suggest that a successful invasion is associated to the generation of a specific displacement of the surrounding matrix.

The human embryos retain their spherical TE or syncytiotrophoblast organization in contrast to alternative 2D setups using glass surfaces, where a more cylindrical embryo organization has been reported with hCGβ- or HLA-G–positive cells localized at the rim of the embryo (*37*). The maintenance of the 3D embryo volume allows us to complement the current picture of human peri-implantation embryo by shedding light on the *z* dimension (Fig. 4, C to E). Our results show how TE differentiation takes place at the interface with the matrix leading to a spherical distribution of TE and syncytiotrophoblast cells located underneath the epiblast compartment.

Overall, our results reveal that human embryos exert dynamic pulling forces on the ECM that propagate in the matrix leading to matrix remodeling and radial displacement patterns. Invasion is associated to a specific matrix displacement highlighting the significance of these forces in the implantation process.

### Force is applied via focal adhesion and podosome-like structures

**Fig. 5.**
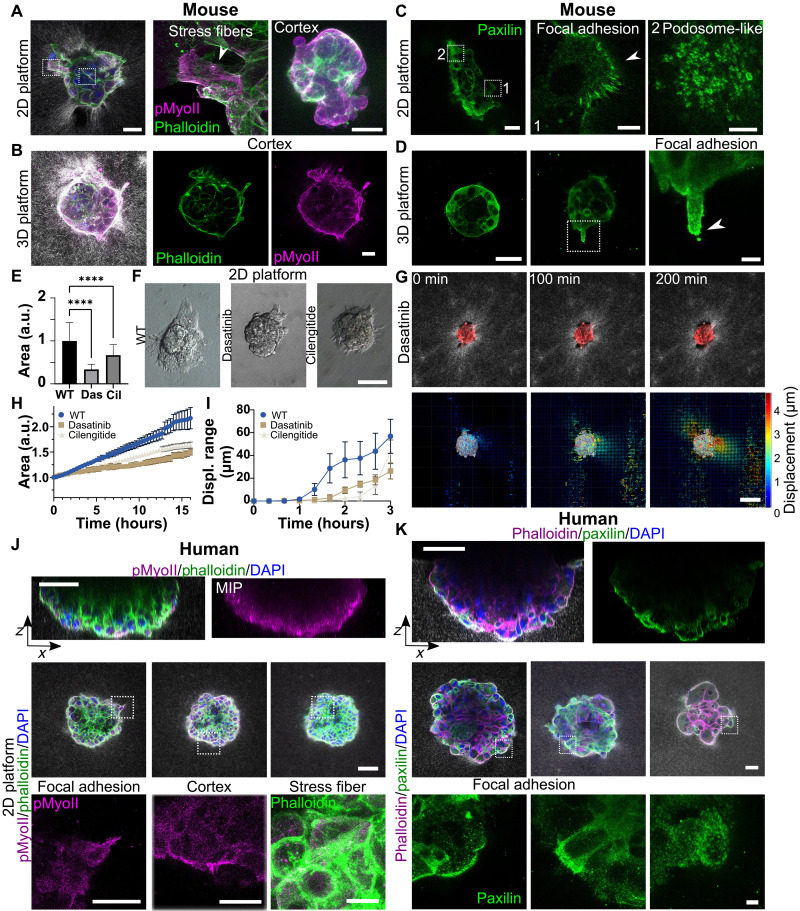
Focal adhesion and podosome-like structures during implantation. (**A** and **B**) Immunostaining of DAPI (blue), phalloidin, and phosphorylated myosin II (pMyoII) in a mouse embryo implanting in 2D and 3D platforms. Inset: Stress fibers and cortex. Scale bars, 50 μm. (**C** and **D**) Paxillin staining of embryos in 2D and 3D platforms. Insets: Podosome-like structures and focal adhesions in 2D (center, right) and 3D protrusions (center, right). Scale bars, 50 and 20 μm (insets). (**E**) Normalized outgrowth size at E7.5 for wild-type (WT) and drug-treated embryos (10 μM dasatinib, 50 μM cilengitide). *N*_WT_ > 3, *n*_WT_ = 109; *N*_Das_ = 3, *n*_Das_ = 51; *N*_Cil_ = 2, *n*_Cil_ = 35. *t* test, ****P < 0.0001. Error bars: SD. (**F**) Snapshots of drug-treated embryos on a 2D platform showing reduced outgrowth. Scale bar, 100 μm. (**G**) Time lapse (single *z* plane) of 10 μM dasatinib-treated embryos (tdTomato, red). Matrix fibers and deformations were captured via light scattering. Bottom: 3D cumulative collagen displacement maps, color-coded by amplitude with displacement direction (arrows) of the entire 3D volume. Scale bar, 100 μm. (**H**) Normalized area variation over time for WT, cilengitide-, and dasatinib-treated embryos. *N*_WT_ = 3, *n*_WT_ = 9, *N*_Das_ = 2, *n*_Das_ = 7, *N*_Cil_ = 1, *n*_Cil_ = 4. Error bars: SEM. (**I**) Graph showing average median distance from embryo boundary (normalized by size) where 3.4-μm displacement occurs for WT, cilengitide-, and dasatinib-treated embryos. *N*_WT_ = 2, *n*_WT_ = 4, *N*_Das_ = 2, *n*_Das_ = 5, *N*_Cil_ = 1, *n*_Cil_ = 4. Error bars: SEM. (**J**) Side and top views of immunostained human embryos (DAPI, blue; phalloidin; phosphorylated myosin) in a 2D platform (D10). Insets highlight cortical myosin and stress fibers. Representative of five embryos. Scale bars, 100 and 50 μm (insets). (**K**) Side and top views of paxillin-stained embryos in a 2D platform (D10). Insets show paxilin structures and focal adhesions. Representative of six embryos. Scale bars, 50 and 10 μm (insets).

To identify potential mediators of the force application, mouse and human embryos were stained for actomyosin and focal adhesion protein paxillin (*50*). Actin and phosphorylated myosin, both main force generators in cells, were enriched in the mouse embryo cortex in both setups (Fig. 5, A and B). Stress fibers formed during the trophoblast outgrowth (Fig. 4A). The mouse embryos on 2D platforms formed focal adhesions at the edges of the outgrowth and podosome-like structures with rings of paxillin covering the whole outgrowth area, as reported previously for TGCs (Fig. 5C) (*51*). Areas of dense podosome-like structures were associated to holes in the collagen scaffold (fig. S6, A and B). These structures seemed to grow into the matrix as the basal side of the outgrowth is not smooth but shows micrometer-sized structures (fig. S6, A and B). In the 3D platform, the focal adhesions were concentrated on the tip of protrusions (Fig. 5D). To probe the role of force transmission, we exposed embryos in a 2D platform to dasatinib (Fig. 5, E and F, and movie S5) an inhibitor of src kinase which acts downstream of integrins. The embryos showed limited outgrowth with reduced implantation kinetics and less collagen displacement compared to untreated controls (Fig. 5, G to I). Blocking the activation of β5 and β3 integrins with a cyclic arginyl-glycyl-aspartic acid (RGD) pentapeptide (cilengitide) also reduced the size of the outgrowth (Fig. 5, E and F) and reduced the reach of the collagen displacement (Fig. 5H and fig. S6C). These results suggest that the force transmission through the focal adhesion machinery was required for the mouse embryos to expand the outgrowth on the collagen surface, thus for implantation.

Actin and phosphorylated myosin could be observed in the cortex of human embryos implanted on 2D platforms and in protruding cells (Fig. 5J). Stress fibers were also visible, however less developed than in human embryos spreading on glass surfaces (fig. S4H). Focal adhesions could be identified in protrusions by paxillin staining, especially in cells connecting to the collagen surface. However, focal adhesions were much less pronounced in the matrix compared to human embryos attaching to the glass surface (fig. S4H). Paxilin structures in the cortex of basal cells seemingly forming a mesh were visible. The later structure may help syncytiotrophoblast cells invade (Fig. 5K). Overall, the presence of focal adhesion and podosome-like structures in TGCs and syncytiotrophoblast, both in contact with the ECM, coupled with the limited outgrowth size upon the inhibition of integrins or downstream signaling in mouse embryos, indicates an active role of integrins in implantation progression.

### Embryos establish mechanical connections during implantation

**Fig. 6.**
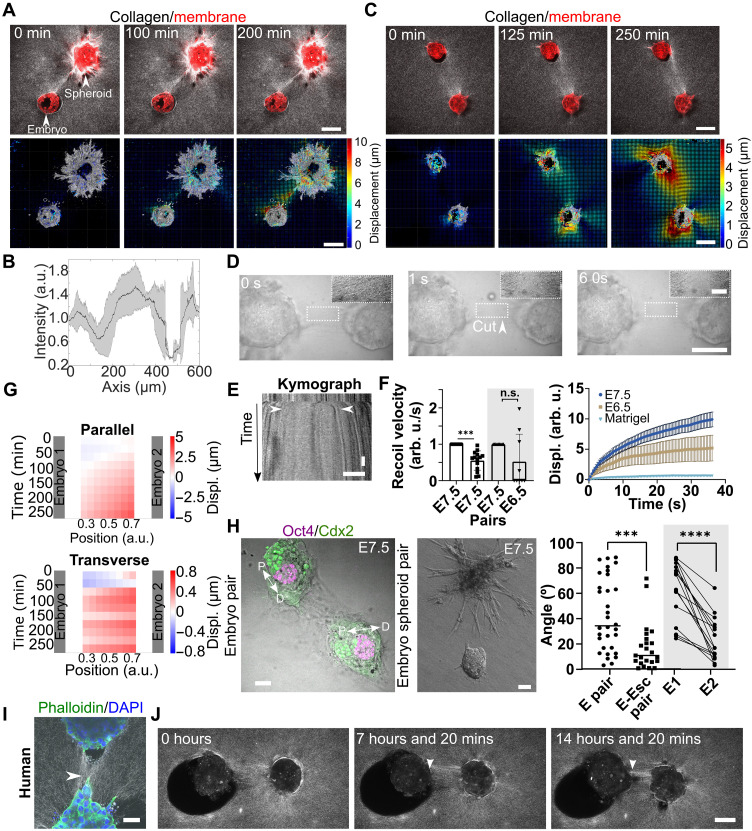
Embryos establish mechanical connections during implantation. (**A** and **C**) Top: Single *z*-plane time-lapse of a mouse embryo with (A) an ESC spheroid or (C) another embryo (tdTomato, red). Light scattering shows collagen fibers and deformations, with increased intensity forming a mechanical bridge. Bottom: 3D cumulative collagen displacement maps show increased displacement along the connecting axis, forming a mechanical bridge. Displacement amplitude is color-coded, with direction indicated by arrows. Scale bars, 100 μm. (**B**) Average intensity along the embryo-spheroid axis. Signal increases between pairs. *n* = 5 pairs, *N* = 2. Error: SD. (**D**) Bright-field time-lapse images of matrix laser ablation. Recoil follows dissection (insets). Scale bar, 10 μm (inset). (**E**) Kymograph of laser ablation, with recoil marked by white arrows. Scale bar, 5 μm, time: 1 min. (**F**) Left: Recoil velocity for embryo pairs; higher toward one embryo. *N* = 4, *n* = 28; Wilcoxon test, *P* < 0.005. Right: Displacement after laser cut: E7.5 versus E6.5 pairs (*n* = 18) and Matrigel pairs (*n* = 6). E7.5 embryos show greater recoil; no recoil in Matrigel. (**G**) Top: Heatmap of collagen displacement parallel and (bottom) perpendicular to the interembryo axis. Initially, collagen moves toward both embryos before favoring one direction. Red indicates positive displacement and collagen densification. (**H**) Snapshot of an embryo pair with labeled epiblast (Oct4 and Cdx2). Double arrowhead shows the P-D axis. Middle: Embryo-ESC spheroid pair in a 3D platform (hanging drop method). Right: Angle distribution of embryo axis relative to the connecting axis: embryo-spheroid pairs (23° ± 4°, ***P < 0.001; ****P < 0.0001, *t* test) and embryo pairs (63° ± 6° and 18° ± 4°, *P* < 0.001, paired *t* test). Spherical embryos were excluded [*n*_sph_ = 16 (*41*)]; minimum ellipse ratio = 1.15. *P* < 0.001, *N* > 3. (**I**) Phalloidin/DAPI staining and (**J**) single *z*-plane time-lapse images of human embryo pairs (D9) with increased collagen intensity (over time) in the 3D platform (*N* = 3). Scale bar, 100 μm. All other scale bars, 50 μm. n.s., not significant.

To investigate how embryos react to external force cues, we placed blastocysts pairwise with spheroids formed by either endometrial stromal cells (ESCs) or 3T3 fibroblasts at a controlled distance in the 3D platform (Fig. 6A). They increased collagen signal (Fig. 6B; fig. S7, A and B; and movie S6) as reported for single cells in collagen (*52*) or as observed when placing spheroids at a similar distance (fig. S7D). However, increased collagen signal was not observed for spheroids formed with T47D cells although they were invasive on glass (fig. S7E). The emergence of these mechanical connections suggests that embryos can feel an active force in their vicinity.

However, spheroids formed by mesenchymal cells would not stay spherical, but cells would migrate into the collagen matrix generating a more diffuse displacement pattern and spheroids maintaining their shape would only poorly engage with the collagen matrix (fig. S7C). To concentrate the force locally in the matrix, we replaced the spheroid by a blastocyst to generate a counter force. Here as well, we observed mechanical coupling, collagen intensity increase, and alignment of the collagen fibers (Fig. 6C; fig. S8, B and C; and movie S7). Similarly, collagen enrichment could be observed on the 2D platform when blastocysts were implanting in the vicinity (fig. S8A and movie S8).

To confirm that collagen in the bridge was under tension, the fibers were severed using laser ablation (Fig. 6D and movie S9). The laser cut was followed by a collagen recoil which was observed toward both embryos (Fig. 6E), confirming that collagen was under tension and that both embryos were pulling along the interembryo axis suggesting a mechanical coupling that we term mechanical bridge. The recoil speed was asymmetrical for the two sides of the cut (Fig. 6F), in agreement with a temporal mismatch of implantation. Performing the laser cut on embryos at different developmental stages [embryonic day 6.5 (E6.5) versus E7.5] reveals an asymmetry of the recoil with embryos of E7.5 showing a higher recoil. Nevertheless, there were some E6.5 embryos that catched up E7.5 embryos and generated more tension reflected in a higher recoil speed (Fig. 6F). This indicates that local fluctuations may allow some embryos to compensate for differences in developmental timing. The recoil speed for individual embryos varied depending on the location of the ablated area because of their anisotropic implantation in the matrix. The range of the released tension was similar for single embryo and mechanical bridge (fig. S8, D and E).

The establishment of the mechanical interaction was sensitive to the initial conditions (fig. S6, F and G): (i) when the initial traction was applied toward each other, a mechanical connection formed quickly, regardless of the distance; in other cases, either (ii) the connection formed at a later time point or (iii) none was established. At the onset of implantation, the blastocyst traction could be described as a tug of war with displacements in the connecting volume taking place toward both embryos. Thereafter, the displacement shifted nearly exclusively in the direction of one embryo of the pair (Fig. 6G and fig. S8H), leading to a displacement gradient, in agreement with the asymmetrical recoil speed observed for blastocyst pairs. The DVC analysis further showed that the fiber densification could be related to the transverse movement of the collagen (Fig. 6G and fig. S5I).

As Matrigel is used in peri-implantation studies, we investigated whether we could observe mechanical coupling on these substrates. However, no increased Matrigel density nor recoil upon laser dissection could be observed (Fig. 6F and movie S10). Nevertheless, upon addition of collagen (20%), mechanical coupling could be recovered (fig. S3G).

To evaluate the impact of external forces on embryo morphology, the axis orientation of each embryo was measured in embryo-spheroid pairs. Embryos oriented their P-D axis (Fig. 6H and fig. S9A) according to the connecting axis (18° ± 4°). The axis orientation was also measured in embryo pairs of elongated shape (threshold ellipse ratio, 1.15; 16 of 41 embryos) and axis parallel to the surface. The angles of each embryo pair were sorted into larger and smaller angles. One embryo assigning #1 was oriented with a 63° ± 6° angle to the connecting axis and the second one assigning #2 with an angle of 23° ± 4° (Fig. 6H) relative to the interembryo axis. Also, transverse movement of the embryo after tension release through laser cuts (fig. S9B) could be observed, suggesting a torque. Together, the results indicate that external force cues could influence the spatial orientation of the forming body axis.

A mechanical connection was also established when placing human blastocysts at a controlled distance (Fig. 6, I and J, and movie S11). Cells sending protrusions were found at both ends of the aligned collagen. The time-lapse movie revealed mechanical bridge formation with a progressive fiber density increase along the interembryo axis. These results strongly suggested that human embryos also retained the ability to apply and sense forces and to establish mechanical interactions during implantation.

Overall, these findings highlight the mechanical connection of the embryos with their environment during implantation, which, in the case of the mouse embryo, can affect the orientation of the body axis formation.

### Embryos sense and respond to an external force cue

**Fig. 7.**
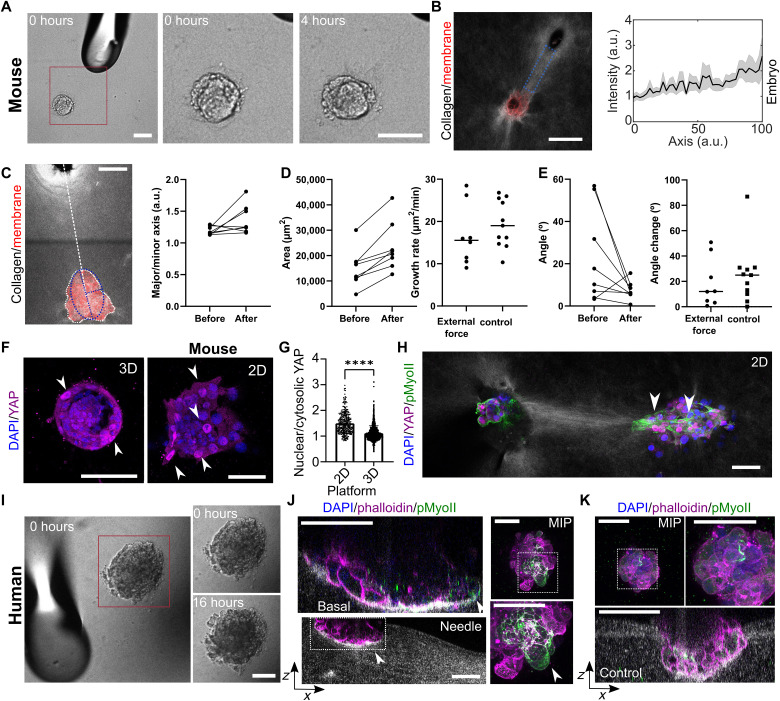
Embryos sense and respond to an external force cue. (**A**) Time-lapse images of microneedle applying pressure next to a mouse embryo in a 2D platform. (**B**) Left: Light scattering of collagen densification between embryo (tdTomato, red) and needle. Right: Normalized collagen intensity along the microneedle (left) and embryo (right) axis (normalized length), collagen intensity increases. *n* = 6 embryos. (**C** to **F**) Embryo area size, morphology, and orientation relative to the connecting axis before and after pressure, compared to controls. Paired *t* test, n.s. (C) Left: Schematic of ellipse fitting (blue) to embryo outgrowth (white) and connecting axis. Right: Outgrowth elongation increases, with the major/minor axis ratio changing from 1.20 ± 0.02 to 1.34 ± 0.09. (D) Outgrowth size increases from 16,472 ± 2531 μm^2^ to 24,958 ± 3521 μm^2^, growth rate similar to control embryos. *N*_WT_ = 4, *n*_WT_ = 11. (E) Embryo alignment with the needle increases, with the average angle decreasing from 22.8° ± 9.0° to 7.1° ± 1.7°. Control embryos show more angle change. *N*_WT_ = 4, *n*_WT_ = 11; *N* = 7, *n* = 8. Error: SEM. (F) Immunostaining of DAPI and YAP in E7.5 mouse embryos on 2D and 3D platforms. YAP is nuclear in some (3D) mural trophoblast cells and (2D) TGCs. (**G**) Plot of nuclear versus cytoplasmic YAP distribution, with a higher ratio in TGCs. *N* = 2, *n*_2D_ = *n*_3D_ = 9. (**H**) Immunostaining of DAPI, phalloidin, and YAP in embryos (2D platform). YAP is expressed in the right embryo along the mechanical bridge. (**I**) Time-lapse images of a microneedle applying pressure next to a human embryo on the 2D platform. (**J** and **K**) Side view and MIP of D10 human embryos exposed to external force or control, stained for DAPI, phosphorylated myosin 2 (pMyoII), and phalloidin. pMyoII is enriched at the basal side (arrows). Representative of *N* = *n* = 3. Scale bars, 100 μm.

To further explore what determines the response of embryos to external forces and how it may affect implantation, we varied the external force source and direction in a controlled manner. To exclude chemical signaling when sensing external mechanical forces, a pure mechanical stimulus was applied in the 3D platform. First, we introduced a rigid boundary constraint, such as blastocyst-sized microbeads or polydimethylsiloxane (PDMS) walls, and observed that this was insufficient to trigger a response of the embryo in form of a collagen densification, direct displacement, or directed embryo invasion (fig. S9, C to E). Therefore, we exposed the TGCs forming the mouse embryo outgrowth to an active external force mimicking fluctuating forces generated by cells by pressing down the matrix with a microneedle in the 2D platform and cyclically varying this pressure (Fig. 7A, fig. S10A, and movies S12 and S13). The needle compressed the matrix generating local collagen densification and geometrical depression, exposing the embryo both to matrix displacement and remodeling which are intrinsically linked. A mechanical bridge formed between the embryo and the microneedle, and the growth direction was (re)oriented to the microneedle tip (Fig. 7, B to E). This finding supports the hypothesis that external forces could be sufficient to trigger embryo response allowing the embryo to modulate the direction of force application during implantation.

To better understand how the mouse embryo senses tension, we stained for the mechanosensitive transcriptional regulator YAP (*53*, *54*). The number of YAP active cells and the nuclear-to-cytoplasmic ratio were higher for the TGCs of embryos implanted in the 2D platform than for mural TE of those implanted in the 3D platform (Fig. 7, F and G). TGCs showed nuclear YAP localization throughout the outgrowth but in one case, a directional localization of TGCs with nuclear YAP signal was observed for embryo pairs (Fig. 7H) or after external force application (fig. S10B). Overall, these experiments suggest that TGCs are subjected to and respond to external forces potentially conferring them a role as drivers for mechanical interaction of the embryo with its environment.

To elucidate the reaction of implanting human embryos to external force cues, they were exposed to a cyclic microneedle pressure on the collagen matrix (Fig. 7I and movie S14). The human embryos reacted to the external cue by forming cell projections toward the microneedle. Phosphorylated myosin 2 was enriched at the basal side of the implanting embryo (Fig. 7J and fig. S10, C and E) suggesting myosin 2 activation. Conversely, control embryos not exposed to external force did not show myosin enrichment or directed cell projection (Fig. 7K and fig. S10D). These results suggest that human embryos could also sense and respond to external force cues. Together, our results hint to a mechanosensitive response of mouse and human embryos influencing implantation orientation.

## DISCUSSION

We used a new ex vivo implantation platform amenable to high-resolution microscopy that enables traction force microscopy of live mammalian (mouse and human) embryos to research the role mechanical forces and mechanosensitivity play in embryo implantation. The embryos preserve the physiological expression of lineage markers consistent with recent reports for extraembryonic and embryonic tissues, supported the formation of the proamniotic and prospective yolk sac cavities and the maintenance of spherical morphology upon implantation. The TE cells that in contact with the matrix expressed TE markers and differentiate to syncytiotrophoblast cells in both setups (*36*–*38*).

Alternative setups such as 3D scaffolds, Matrigel addition to culture, and endometrial organoids have been recently used, respectively, to study mouse (*47*) and human (*38*) embryo development and interaction with endothelial (*55*) and endometrial stromal cells (*56*). However, our method made it possible to quantify the implantation dynamics and determine its mechanical footprint by direct imaging of the fibers and quantification of displacement through DVC. Both mouse and human blastocysts applied forces to invade the matrix; however, mouse embryos showed anisotropic displacement pattern with two to three robust axes of displacement, whereas human embryos started with a more discrete displacement before switching to multiple small foci pulling radially over time. The displacement directions generated by mouse embryo were more stable over time. The DVC analysis revealed a fluctuating pattern for both species, which may suggest a scanning mechanism of the mammalian embryo (*49*). Mouse embryo outgrowth was limited to the surface of the matrix, whereas human blastocysts integrated into the matrix through the polar trophoblast side, consistent with species specific implantation differences in vivo (*12*, *13*, *57*, *58*).

We could tune mouse invasion behavior by varying matrix stiffness and composition, observing a minimal outgrowth formation with a maximum implantation depth for Matrigel. However, we could not vary matrix stiffness, ligand density, or viscoelasticity independently of one another. Matrigel has so far been used as a surface coating for embryo implantation, in combination with collagen (10%) in implantation setup (*47*) or for human embryo culture in suspension (*38*). We could speculate that increasing gel stiffness would lead to more spreading of the human embryo on the surface and a reduced invasion depth as it is the case on rigid glass substrate where an outgrowth of the human embryo can be observed. Vice versa, limiting the human embryo invasion depth may trigger more spreading on the surface of the gel. Matrix stiffness and viscoelasticity have been shown to influence spatiotemporal growth of encapsulated breast cancer spheroids and trigger symmetry breaking leading to tissue branching (*59*). The relation of invasion depth versus embryo spreading and the exact role of stiffness, ligand density, and matrix viscoelasticity on the invasion characteristics needs to be determined in future studies.

The origin of the species-specific implantation characteristics that we observe in vitro and that have been reported in vivo is poorly understood. Our results show that mouse embryos can switch to integration in an ECM depending on the matrix composition. Human embryos may apply higher forces compared to mouse in absolute terms; however, normalizing the displacement by size shows an increased displacement per contact area for mouse embryos. We could speculate that the asymmetric cell number of mural to polar (higher for mural) TE and subsequent differentiation as human embryo implant via the polar TE and mouse embryo via the mural TE (*60*) may explain the differences in traction amplitude or that the ligand availability could set the symmetry breaking (surface versus depth implantation). In vivo mouse embryos get engulfed by the tissue forming a decidua (*12*, *13*); therefore, the tissue needs to bend around the blastocyst to which it may mechanically contribute by forming some stable displacement directions. Mutants of Vang-like proteins 2 led to defective planar cell polarity signaling with defective crypt formation and impaired pregnancy outcome (*61*). It would be interesting to determine whether the displacement patterns we observe during implantation are conserved, in other mammalian species—such as cow, marmoset, or rabbit—particularly comparing those with interstitial implantation (like humans) to those with superficial implantation (like mice).

Perturbing src kinase downstream of integrin signaling or directly integrins with a RGD pentapeptide resulted in restricted implantation in mouse embryos. Integrin knockout mutants have been reported to (*62*) show a limited expansion of the TE on surfaces (*62*). Consistent with this, reduced collagen displacement was associated to poor implantation progression, establishing a link between matrix remodeling and implantation capacity in the human embryo.

Our observations are in agreement with a mechanosensitive behavior of the peri-implantation mouse and human embryo. Mechanosensitivity has been suggested to play a role in cell lineage segregation at the blastomere stage for mouse and human embryo (*24*, *63*). In our system, for the mouse embryo, several observations support the mechanosensitive capacity of the embryos: (i) the modulation of YAP localization [a mechanosensitive transcriptional regulator (*53*, *54*)] in the nuclei of mouse trophoblast; (ii) the mouse embryo orientation affected by external force cues in both platforms. In addition, mouse embryos also showed a preferential growth direction toward their main pulling axis in the 2D platform, suggesting a mechanosensitive feedback loop. In the case of human embryos, the mechanosensitive behavior of the peri-implantation embryos was supported by their reaction to the external force cue by forming directional cell projections and accumulating phosphorylated myosin 2 at the basal side of the embryo. Collectively, these findings suggest a mechanosenstive behavior of the peri-implantation mammalian embryo and a potential role of external mechanical forces in guiding embryo implantation.

Similar mechanical interactions through matrix bridges have been observed in other biological systems, including individual cells (*52*), cell spheroids, mammary acini, or the attraction of macrophages by contractile fibroblasts in collagen matrices (*64*–*66*). We observed mechanical interactions for spheroids formed by different types of fibroblasts; however, a more epithelial cancer cell type, despite being invasive, did not show this type of interaction. A requisite for mechanical bridge formation may be epithelial to mesenchymal transition or polarity inversion as reported for mouse embryos (*67*, *68*). In addition, these interactions are facilitated by the nonlinear properties of the fibrous matrix, such as fiber buckling and stiffening (*43*). The mechanical properties of the endometrial matrix may also favor the long-range transmission of forces because of the nonlinear response of collagen network fibers (*40*).

When implanting in the uterus, the blastocyst faces a complex mechanical environment. Uterine tissue rearrangements occur in the uterus at the time of implantation (*69*), and primary TGCs are exposed to oscillating intrauterine pressure (*30*, *70*). We observed an orientation of the mouse P-D axis. This could indicate a direct link between the establishment of the developmental axis in the mammalian embryo and the external mechanical environment. In vivo alignment mismatch leads to mid-gestation lethality and compromises pregnancy outcomes for mouse embryos (*71*), underscoring the importance of embryo orientation and the potential role of external forces in this process. We speculate that the trophoblast can act as a mechanical coupling system, connecting the maternal mechanical environment with the embryonic tissues. In agreement with this hypothesis, external force cues have been suggested to contribute to the in vitro formation of the A-P axis in postimplantation mouse embryos (*29*, *70*). However, how the invasion led by the trophoblast affects human epiblast polarity or development needs to be further determined in future studies.

In vivo, the mouse embryo aligns its P-D (also referred to as embryonic-abembryonic) axis with the mesometrial-antimesometrial axis (*69*). Likewise, the endometrium thickening and the contractions of the myometrium have been proven to contribute to embryo positioning in equines (*72*) and mouse (*31*), which supports the hypothesis that the mechanical environment could orient the implantation of mammalian embryos. The human uterus exhibits spontaneous contractions with frequencies of 1 to 1.6 contractions/min, which vary throughout the phases of the menstrual cycle (*73*). Consequently, human embryos may be exposed to these uterine contractions and the associated forces during implantation. Patients with in vitro fertilization (IVF) and a positive pregnancy outcome have similar contraction frequencies as women in the lutheal cycle, whereas women with a negative pregnancy outcome have a reduced uterine contraction frequency at the day of embryo transfer (*74*). By days 5 to 6 after embryo transfer, uterine contraction frequency was higher, and amplitude of contraction was reduced in patients with ongoing pregnancies (*74*). High frequency of uterine contraction (>2 contractions/min) on the day of embryo transfer in patients with IVF showed reduced pregnancy outcomes and implantation rates as higher frequencies may expel embryos from the uterus (*75*–*77*). This suggests that there may be an optimal frequency range favorable for embryo implantation (*74*). These findings highlight the importance to understand both (i) the forces the human embryo applies and (ii) the response of the embryo to external forces.

We acknowledge that our cell-free setup does not comprehend the whole cellular complexity of the uterus (*71*, *78*, *79*) or the ECM composition (*80*, *81*); however it allows identifying the embryo-specific contribution to implantation and facilitates mechanical studies. Together, our in vitro work revealed the mechanical signature of embryo implantation, the mechanosensitivity of mouse and human embryos, and the impact of mechanical forces in embryo implantation. The source of mechanical cues and their role in steering implantation in vivo remain an open question to be addressed in the future.

## MATERIALS AND METHODS

### Mouse embryo recovery and culture

Animal Care and Use committee CEEA-PCB-OH (Comitè Ètic d’Experimentació Animal del PCB - Òrgan Habilitat) and the regional Government (Generalitat de Catalunya, committee approval number 11338) approved animal experiments. Four- to 6-week-old female mice were superovulated with PMSG (pregnant mare serum gonadotropin) by injection of 5 IU intraperitoneally and a second injection of hCG at 46 to 48 hours. Afterward, the females were mated with males. The next morning, the mice were checked for vaginal plug (E0.5), and one-cell stage embryos were collected in M2 media (Merck, M7167). Embryos were cultured until blastocyst stage in drops of 25 to 50 μl of KSOM Advanced medium (Merck, MR-101-D), and they were covered with mineral oil (Nidacon, NidOil) and incubated at 37°C, at 6% CO_2_ and 5% O_2_. The mT/mG (RRID:IMSR_JAX:007676) mouse line was used for time-lapse experiments and C57BL/6J (RRID:IMSR_JAX:000664) or B6CBAF1/J (RRID:IMSR_JAX:100011) otherwise.

The composition of the IVC1 medium was previously described (*82*), consisting of Advanced Dulbecco’s modified Eagle’s medium (DMEM)/F-12 (Life Technologies, 12634-010), supplemented with 20% heat-inactivated fetal bovine serum (FBS; Thermo Fisher Scientific, 10270106), 0.5× penicillin (25 U/ml)/streptomycin (25 mg/ml) (Life Technologies, 15070-063), 2 mM l-glutamine (Life Technologies, 25030-024), 1× ITS-X (Life Technologies, 51500-056), 8 nM β-estradiol (Merck, E8875), progesterone (200 ng/ml; Merck, P6149), 1 mM sodium pyruvate (Merck, 11360-070), and 25 μM *N*-acetyl-l-cysteine (Merck, A7250-25g). For IVC2 medium, FBS is replaced by knockout serum (Life Technologies, 10828-010) at 30%.

### Human embryo donation and ethics approval

All human embryos used in this work were donated from IVF treatment surplus. All donor couples provided their signed informed consent and received the corresponding letter of information of the research project. The embryos used were cryopreserved at different stages of development and donated for research following Spanish Law 14/2006 of Assisted human reproduction. All embryos used in this work were donated from Dexeus Mujer. The embryos were assigned to project IMPLANT-Seq (committee approval number 19-01_PI-CAT PI 2019_10), to project SeraSupp [committee approval number DGP 2015_27 (#0336S/2709/2016)], and to project HYSPLANT (committee approval numbers 21-06_PI-CAT PI 2021_44). All embryos were destroyed once the research was finished and were not maintained in culture longer than day 14th of development after fertilization following National Law 14/2006. All samples obtained from patients were anonymized at the donation center.

### Human embryo culture

Cryopreserved days postfertilization 3 and 5 embryos were thawed using a Kitazato Thawing kit (VT602, Kitazato) according to the manufacturer’s instructions. Once the thawing was complete, the embryos were cultured in preequilibrated either Global Total LP culture media (HSGT-010, Origio Medicult) or Global (LGGG-020, Origio Medicult) supplemented with Plasmanate (GRIFOLS) or HSA [albumin (human) U.S.P. Human Albumin Grifols NDC61953-0001-2] at 37°C, 6% CO_2_ and 5% O_2_. Embryos were morphologically scored upon thawing and cultured until day 6. Embryos were transferred to transfer medium containing embryo transfer supplement (ETS; patent: EP3696260A1, GRIFOLS) where they stayed for 4 to 6 hours before zona was removed using acidic Tyrode’s solution (Merck, T1788). Embryos were then placed on a 2D or 3D platform with preequilibrated IVC1 medium supplemented with FBS, HSA, or ETS at 20%. In a 3D platform, the medium was switched to IVC2 at D7 or D8 and in 2D platform at D9. At the end of the experiment, the embryos were fixed using formalin.

### Endometrial stromal cell extraction and spheroid culture

Uteri were removed from 3- to 4-week-old mice, and the organ was cut open longitudinally. Fat tissue and blood vessels were removed, and the uteri were placed in 0.5% trypsin (Life Technologies, 15400-054). After washing with Hanks’ balanced salt solution (Life Technologies, 14174-053), uteri were transferred to 0.05% trypsin and placed at 37°C and 5% CO_2_. The supernatant and uteri were decanted through a 0.4-μm mesh strainer (Cultek S.L.U., 45352340). After various centrifugation steps and removal of red blood cells with lysis buffer (Miltenyi Biotec,130-094-183), the cells were plated with DMEM/F12 (Life Technologies, 21041025) supplemented with 1% penicillin-streptomycin (penstrep; Sigma-Aldrich, P0781-100) and 20% FBS (Life Technologies, 10270098). Cells were resuspended at 25,000 or 50,000 cells (3T3 fibroblasts) or 1 to 2 million (ESCs) in 500 μl of DMEM (Life Technologies, 31331-093) supplemented with 10% FBS and 1% penstrep in a Corning 24 ultralow attachment plate (VWR, 734-1584). 3T3 fibroblasts were cultured in DMEM (Life Technologies, 31331-093) supplemented with 10% FBS and 1% penstrep. To obtain a regular distribution, we followed Ergaz *et al.* (*83*). T47D cells were cultured in RPMI (Life Technologies, 11835030) supplemented with 10% FBS and 1% penstrep. After spheroid formation after 2 to 3 days, they were placed in the 3D platform with embryos.

### Immunostaining

Mouse embryos were fixed after 2 to 3 days of implantation at E6.5 or E7.5 and human embryos between D9 and D12 with formalin (Merck, HT5011-1CS), washed with phosphate-buffered saline (PBS; Life Technologies, 10010-015), and stored at 4°C. Permeabilization was done using 0.5% Triton X-100 (Merck, T8787-250ML) and blocking with blocking buffer [1% bovine serum albumin (BSA) (Merck, A7906-50g) + 3% donkey serum (Merck, S30-100ML) + 0.3% Triton X-100 in PBS]. Then, primary antibodies were incubated in primary antibody buffer (0.1% BSA + 0.3% donkey serum + 0.2% Triton X-100 in PBS) and secondary antibodies in secondary antibody buffer (0.1% BSA + 0.3% donkey serum in PBS). The following primary antibodies were used: phospho-myosin light chain 2 (Ser^19^) (1:100; Cell Signaling Technology, #3671), paxilin (1:200; Abcam, ab32084), YAP (1:100; Santa Cruz, sc-101199), phalloidin 488 (1:100; Thermo Fisher Scientific, A12379), 4′,6-diamidino-2-phenylindole (DAPI; 1:500; Thermo Fisher Scientific, D1306), phalloidin 568 (1:100; Thermo Fisher Scientific, A12379 ), Cdx2 (1:500; Abcam, ab76541), Oct4 (1:200; Santa Cruz, sc-5279), laminin (1:200; Abcam, ab11575), and PARD6B (B-10) (1:200; Santa Cruz, sc-166405). In addition, anti–hGata-6 (1:200; R&D Systems, AF1700), CK7 (1:-300; Abcam, ab181598), syndecan-1 (1:100; Abcam, ab128936), HLA-G (1:100; Abcam, ab52455), and hCGβ (1:100; Abcam, ab9582) were used for human embryos. AF647 (the Jackson Laboratory, 715-605-151), AF647 (the Jackson Laboratory, 705-605-147), AF488 (the Jackson Laboratory, 711-545-151), and AF488 (the Jackson Laboratory, 711-545-152) were used as secondary antibodies at 1:500. Mouse embryos in Matrigel culture were fixed using formalin and glutaraldehyde (Merck, G6257).

### Experimental setups

To assemble the 3D platform, a collagen (CellSystems, 5074-35ml) or Matrigel (Corning Matrigel 356231) drop of 50 μl was deposited on a glass-bottom dish (MaTtek, P35G-1.5-14C) avoiding air bubbles, and the embryos (E4.5 or E5.5) were carefully pipetted inside the drop and let polymerize at 37°C for a maximum of 90 min. IVC1 culture medium was added and covered with mineral oil. Embryos were placed individually or in pairs at a distance of 150 to 250 μm. For experimental purpose, collagen drops were polymerized upside down in a “hanging drop” conformation and cultured for 48 hours or the sample flipped back to standard orientation covered with medium and oil. A 2D platform was fabricated by depositing a 50- to 100-μl drop of collagen or Matrigel on a glass-bottom dish. The plate was centrifuged twice at 1200 to 2000 rpm for 3 min with a swinging rotor. Collagen was polymerized at 37°C and 5% CO_2_ for 90 min. Embryos were placed in a drop of 350-μl IVC1 medium on the 2D platform which was then covered with mineral oil. Dasatinib (SML2589, Merck Life Science) was used at a concentration of 10 μM and cilengitide (InvivoChem, MC23042603) at 50 μM. For 2D glass experiments, embryos were cultured on eight-well IbiTreat μ-plates (Ibidi GmbH, 80826).

### hCG quantification

Culture medium of embryos implanting on a 2D platform was collected from D6 to D8. hCG levels were quantified using the Human hCGβ ELISA Kit (EH235RB, Thermo Fisher Scientific) following the manufacturer’s instructions. A standard curve was obtained using a four-parameter logistic regression algorithm. Samples were diluted 30 times, and IVC1 medium was added as a negative control. The absorbance at 450 nm was measured in duplicates for all samples using a Synergy HTX Multimode Reader (BioTek), and the final concentration for each sample was calculated using the standard curve.

### Microfabrication

Polydimethylsiloxane (PDMS; Sylgard) prepolymer and the curing agent were mixed in a 10:1 mass ratio and pipetted during approximately 3 min. The PDMS solution was poured in a petri dish, placed in a desiccator to remove air bubbles, and cured overnight at room temperature. Pieces of PDMS were cut, plasma was activated, as well as MatTek dishes, and bonding was performed. A collagen drop was deposited on the PDMS, and embryos were placed as described above.

### Imaging

Imaging was performed on a Zeiss LSM-780 inverted microscope with a 25× multi-immersion objective [numerical aperture (NA) = 0.8, Plan-Apochromat], a 32× water objective (NA = 0.85, Plan-Apochromat), or a 40× water objective (NA = 1.3, Plan-Apochromat). Signal was collected using a photomultiplier tube or a GaAsP detector at Airy 1. The collagen mesh was acquired in reflection mode using the 488-nm Argon laser. Time-lapse imaging was performed with the 25× or 32× objective. The z-stack typically ranged from 60 to 150 μm with a *z* spacing of 1.4 to 1.8 μm and time between 15- and 25-min frames. Human embryos were either imaged in bright-field or with a multiphoton acquisition, which allows capturing autofluorescence of the embryo. Autofluorescence was induced at 780 nm with 3.5% of a MaiTai laser HP DS of a total power of 3.0 W at 80 MHz (measured at 800 nm) and detected in the range of 350 to 700 nm with nondescanned detectors.

### Laser ablation

Laser ablation was performed on a custom-built laser nanosurgery system, using a train of pulses from a subnanosecond ultraviolet laser (Teem Photonics, France) coupled into the epi-port of an inverted microscope (*84*) (Axiovert 200M, Carl Zeiss Microscopy GmbH, Germany) and equipped with a Nipkow spinning disk without microlenses, fed with 488 and 561 diode lasers (LightHub, Omicron Laserage Laserprodukte GmbH, Germany). Imaging was performed with an ORCA Flashv2 sCMS camera (Hamamatsu, Japan). A line of 50 to 100 μm, with 5 to 6 pulses/μm, was laser cut in the matrix in three planes with a *z* interval of 10 μm. The imaging rate was one to three images/s right after cut and 5 s after 1 min. The cut location was chosen equidistant between two embryos for pairs and at a similar distance for single embryos. Image intensity was normalized, and Laplacian filter was applied to bright-field images. Displacement of collagen along the connecting axis was determined via the KLT tracking algorithm (*85*) originally from H. Delanoë-Ayari (https://github.com/heleneayari/2DTFM) and summed over time. To establish initial recoil velocity, *v*_0_ was calculated from the fitting function *f*(*t*) as follows

$$f\left(t\right)=A_{1}\left[1-\text{exp}\;\left(\frac{t}{\tau_{1}}\right)\;\right]\;\text{and}\;v_{0}=\frac{A_{1}}{\tau_{1}}$$

where *A* denotes the retraction amplitude and τ denotes the time constant of the exponential (fig. S11). For pairs showing lower recoil, displacement was calculated on an interval of 2.5 s to reduce noise. To plot the difference of recoil velocity in embryo pairs, the recoil velocities of each pair were sorted into two groups, either smaller recoil velocity or larger recoil velocity.

### Micromanipulator

Constant or varying pressure was applied next to an embryo implanting on the 2D setup with a microneedle mounted on a microinjector (specs microinjector control). To obtain a varying pressure, the needle was put in a position where it deformed the substrate. This pressure was released for every time-lapse z-stack as the sample was moved down by the Piezo driver. Afterward, it regained its initial position pressuring the hydrogel. The image acquisition was done with a period of 5 to 15 min for mouse and 10 min for human embryos. The microneedle was placed at 250 to 480 μm from the mouse embryo and 400 to 580 μm from the human embryo. The experiment was performed on an Andor spinning disk (Yokogawa, CSU-X1) mounted on an Olympus IX 81 with a 10× objective (NA = 0.3, UPlanFL N) equipped with an iXon EMCCD Andor DU-897 and temperature and CO_2_ control in a whole incubation chamber. Microscope was controlled with Andor iQ software. Constant pressure was obtained by performing a z-stack moving the objectives and oscillatory pressure by using Piezo control, moving the stage for the z-stack. To characterize the collagen densification, image intensities were summed, and the intensity along the connecting axis was determined from the center of the microneedle to the collagen outgrowth interface. The intensity was normalized by the intensity from the center of the needle to a control area where no embryo was located.

### Elasticity measurement

The microrheology was probed with an AFM NanoWizard 4 Bioscience AFM (JPK Instruments) mounted on the stage of an inverted optical microscope (Nikon Eclipse Ti-U). Silicon-nitride V-shaped cantilevers with a constant force of 0.08 N m^−1^, a resonance frequency of 17 kHz, and a cantilever length of 200 μm (NanoWorld Innovative Technologies, PNP-TR-50) were used for the indentation measurements. To obtain the elastic modulus, the Hertz model for a pyramidal tip was fitted to the measured force-distance curves, using the proprietary JPK data analysis software.

### Collagen fibers

Collagen fiber orientation was measured using the ImageJ plugin OrientationJ with sigma 2 or 5 and a pixel range of 10 pixels. The data were further processed in MATLAB (MATLAB 2020) thresholding for low energy and coherency values and removing the embryo signal. The orientation of the fibers relative to the embryo center was determined and plotted in a histogram from 0° to 90°. A 0 difference corresponds to a radial orientation of the vector and 90° to a transverse orientation of the collagen fibers. The numeric order parameter (NOP) was calculated as NOP = <cos 2(φ − ρ)> where φ denotes the angle of origin and ρ the angle of the vector. For the fiber realignment between embryo pairs, the distribution of collagen fibers orientation was analyzed for interembryo space and the control area, and the difference was plotted as a histogram. To measure the collagen intensity around single embryos, a 360° reslice was performed around the embryo center with radial reslice (ImageJ), and the signal was summed up. The intensities were normalized by the signal at the edge of the image. For pairs, the reslice tool was used along the connecting axis of the pair enclosing a thickness of 80 to 110 μm. The signal was normalized by a control region.

### Embryo morphology

Bright-field images of embryos were used to manually outline the area of the embryo which determines the outgrowth size for mouse embryos and whole embryo size for human embryos. To measure the outgrowth direction, images were binarized, and the center of the mass and area was extracted with an ImageJ particle analyzer. An image was cut into 12 equally sized wedges with the center of mass of embryo as the origin. The area was quantified for each wedge, and growth was determined by subtraction of initial area size. The embryo axis was determined by outlining the embryos with a Fiji drawing tool, and their center of mass was determined. The interembryo axis was drawn between the center of mass of both embryos. The shape of the embryonic structure comprising the mural trophoblast, epiblast, and ExE was determined in bright-field images, an ellipse was fitted to the outline (fig. S9A), and the orientation of the major axis relative to the connecting axis was determined. Embryos of spherical shape were discarded; the minimum ellipse ratio was of 1.19 (major/minor axis).

### YAP nuclear localization

YAP and nuclei were costained, and nuclei were detected using StarDist (*86*) and tracked using TrackMate (*87*). Nuclei were expanded, and the cytosol rim was used for normalization. Intensity was measured using MorphoLibJ (*88*), and the ratio of nuclear versus cytoplasmic signal was plotted.

### Statistical analysis

The number of experiments (*N*) and the number of embryos (*n*) included in every experiment are indicated in the corresponding figure captions. Individual data points are shown, when possible, accompanied by the mean value and error bars corresponding to the SEM. The statistical analysis was done with GraphPad Prism; unpaired *t* tests were performed if not stated otherwise; and for sorted data, a pairwise *t* test or Wilcoxon signed rank sum test was applied. The outcomes are shown in the corresponding panels as well as indicated in the figure captions. Cross-correlation analysis was performed on outgrowth direction and collagen displacement. The xcorr function of MATLAB was used with coefficient normalization so that the autocorrelation at zero lag is equal to 1.

### Digital volume correlation

We analyzed a subset time window before the collagen densification saturated the signal or the collagen deteriorated. To compute matrix displacements, reflection images of the matrix were used as a speckle pattern for a DVC algorithm, implemented in MATLAB (MathWorks, R2022b), as previously described (*39*, *89*), originally from Franck *et al.* (*90*) with a DVC code publicly available at https://github.com/FranckLab?tab=repositories. The entire z-stack volume of 6.3 × 10^6^ μm^3^ (560 mm × 560 mm × 200 mm) was segmented into overlapped subsets of 8.8 × 10^3^ μm^3^ (14 μm × 14 μm × 45 μm) with a resolution of 7 μm in the *xy* axes and 22.5 μm in the axial direction and correlated each with respect to the initial time as an unremodeld state (cumulative analysis) or with respect to the previous time point (relative analysis). Displacement magnitudes and direction were calculated for areas around single embryos and in between embryo pairs.

### Direction of displacement

For single embryos, an area around the embryo and excluding the embryo was subdivided to 12 equally sized wedges. Displacement in these subareas was then analyzed and presented either as a single summed up value of displacement values or as a maximal value for average displacement in the sector. These values were then plotted as a heatmap (displacement in a sector versus time). For single embryo and pairs, the displacement in a torus encircling each embryo was extracted and plotted in a polar plot. Tori thickness was half the distance between the two embryos.

### Displacement range

Maximal distance to an observed average displacement larger than a minimum cutoff value is plotted over time. Displacement averaging was performed for each of the 12 sectors around an embryo, and each sector was subdivided to 20 equally wide arches. Displacement average was calculated for every arch.

### Mechanical connections

The displacements between two embryos were quantified in a region of interest between the two embryos. This region of interest had a cylindrical shape, it was as long as the distance between embryos, and the width corresponded to an embryo diameter. Components of displacement vectors were calculated for three canonical spatial directions for cylindrical axes (axial, radial, and tangent) and plotted in a heatmap.
