# Supplementary material for: Traction force and mechanosensitivity mediate species-specific implantation patterns in human and mouse embryos
Source: Sci Adv. 2025 Aug 15;11(33):eadr5199. doi: 10.1126/sciadv.adr5199 (PMC12356271; doi:10.1126/sciadv.adr5199)
Supplement: Supplementary file 1 — Figs. S1 to S11 Legends for movies S1 to S14 [file sciadv.adr5199_sm.pdf]

Science Advances
AAAS

Supplementary Materials for

Traction force and mechanosensitivity mediate species-specific implantation patterns in human and mouse embryos

Amélie Luise Godeau *et al.*

Corresponding author: Amélie Luise Godeau, agodeau@ibecbarcelona.eu;
Ayelet Lesman, ayeletlesman@tauex.tau.ac.il; Samuel Ojosnegros, sojosnegros@ibecbarcelona.eu

Sci. Adv. **11**, eadr5199 (2025)
DOI: 10.1126/sciadv.adr5199

The PDF file includes:

Figs. S1 to S11
Legends for movies S1 to S14

Other Supplementary Material for this manuscript includes the following:

Movies S1 to S14

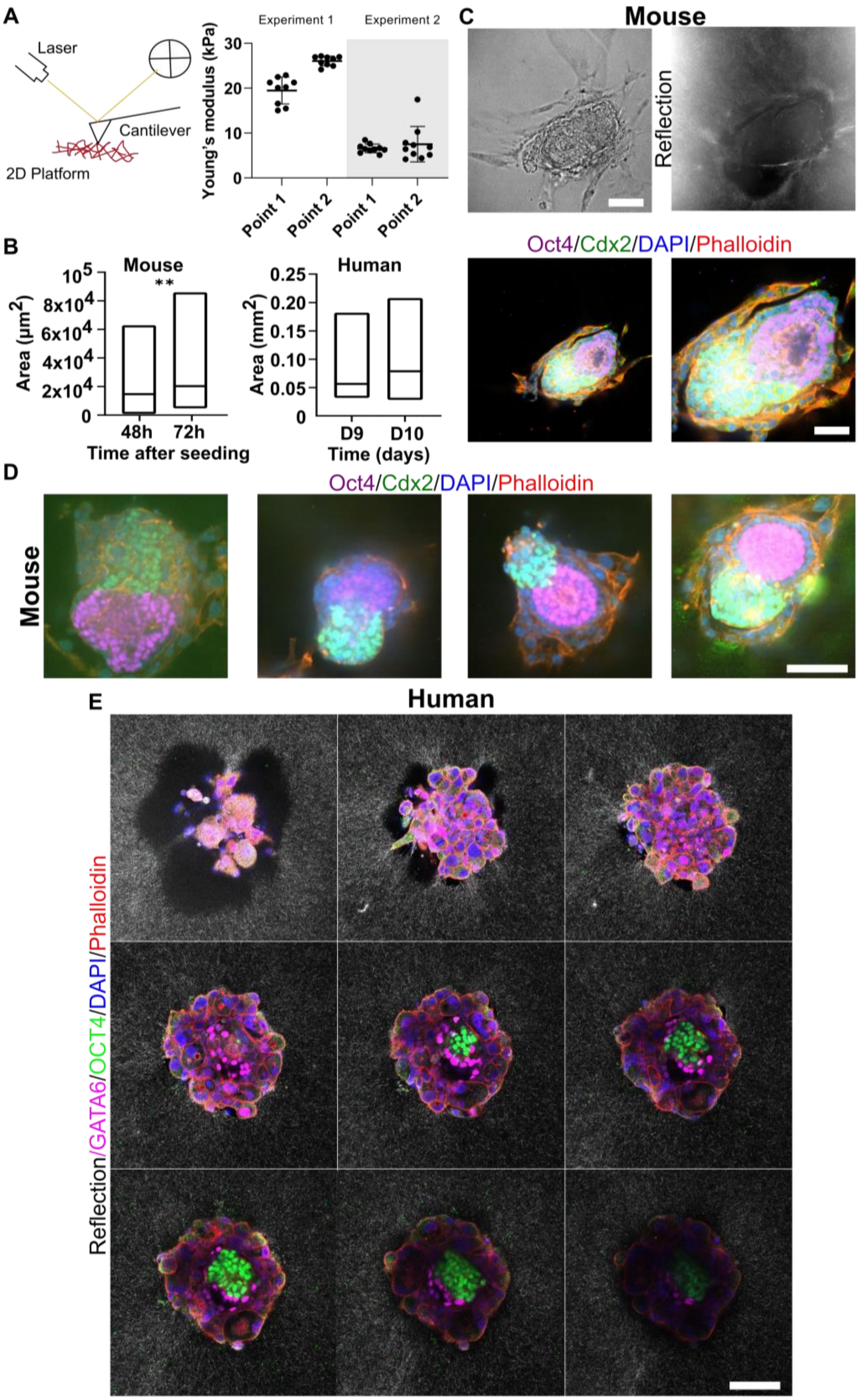

Fig. S1. Mechanical characterization and implantation on the 2D platform. A) (left) Schematics of the atomic force microscopy (AFM) measurement. (right) Distribution of elastic modulus obtained by AFM for 2 points on the 2D platform. N=2. **B)** (left) Area of mouse embryos on the 2D platform 48h and 72h after seeding. Outgrowth area increases from 14931± 938 μm^2at 48h (E6.5) to 21290±1984 μm^2 at 72h (E7.5). n= 50 and N>3. (right) Human embryo size in the 2D platform over time increases from 0.066±0.007 mm^2 at D9 to 0.083±0.007 mm^2. n= 25 and N> 3. The error bar represents the standard error of the mean (SEM). **C)** Snapshot of mouse embryo at E8.5 in 3D platform forming an egg cylinder. Inset

scale bar 50um. **D)** Snapshots of mouse embryo at E8.5 in 3D platform completing egg cylinder formation. Scale bar 50 µm. **E)** Series of confocal z-sections of the D9 human embryo (Fig 1J) stained for OCT4 (green), GATA6 (magenta) with DAPI (blue) and Phalloidin (red). Scale bar=100 µm

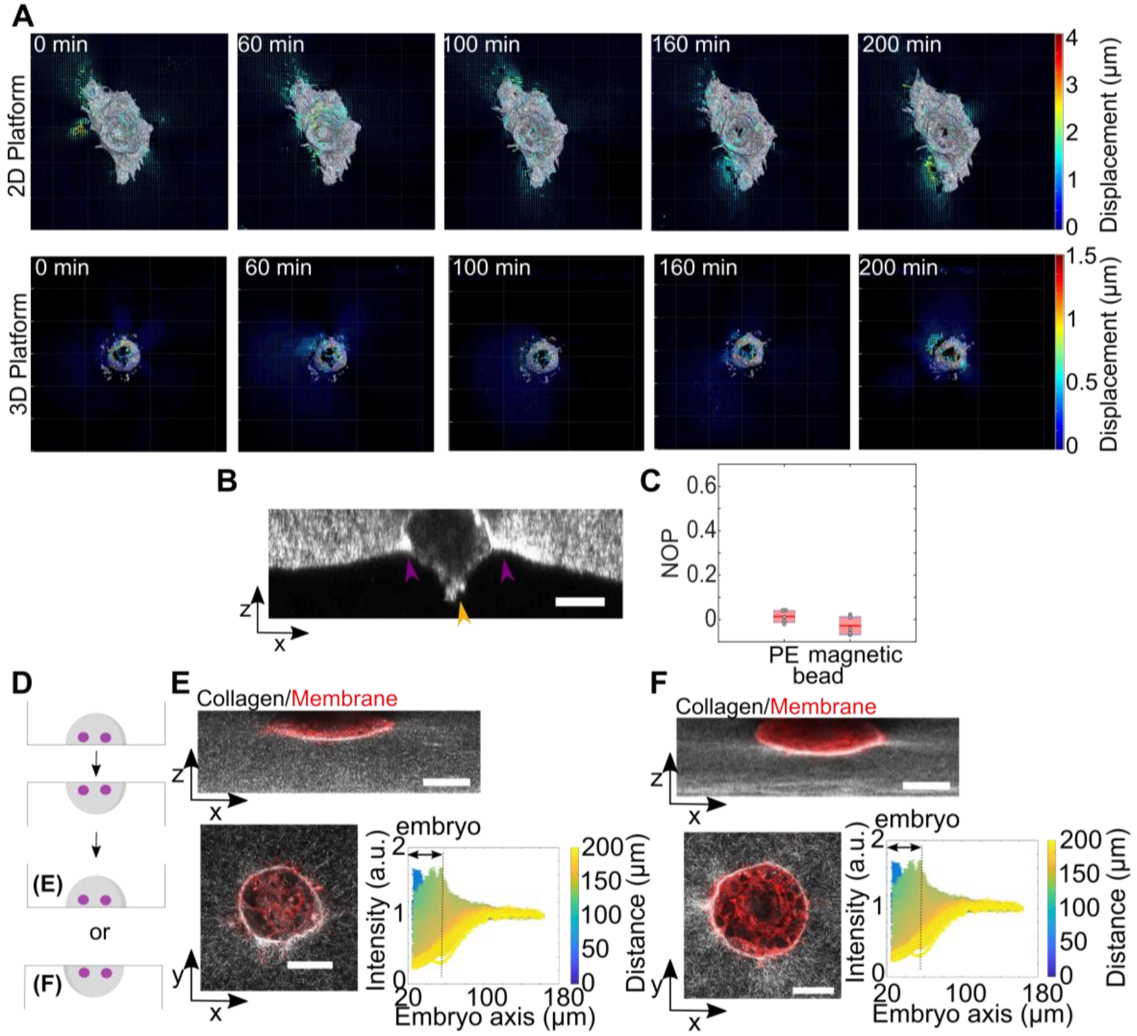

Fig. S2. Mechanics of mouse embryo implantation. A) Map of relative collagen displacement between two consecutive time points generated by single embryos in (top) 2D and (bottom) 3D platforms. The main axis of collagen displacement varies over time. **B)** Snapshot of the side view of light scattering of an embryo embedded in a floating 3D platform. The collagen signal (purple arrows) is higher than the signal of the embryo (orange arrow). Scale bar=50 μm. **C)** Nematic order parameters for embedded beads, polyethylene non-coated and carboxy coated magnetic beads of size 100 μm in collagen. n=5 and N=1, respectively. No order of collagen could be found. **D)** Schematic description of the hanging drop (HD) procedure: Embryos are deposited in a collagen drop and set to polymerize upside down. The culture is maintained for 48h (E7.5), either upright or flipped. **E)** and **F)** Conventional HD collagen polymerization and 48h of upside-down culture respectively. (top) Orthogonal view of an embryo in HD. Scale bar=50 μm. (bottom left) Snapshot of top-view of an embryo in HD. Scale bar=50 μm. (bottom right) Average radial intensity for different z-planes. The Z-position is color-coded. The intensity increases underneath in a rim around the embryo. n=6, N=2, the error bar represents the standard error of the mean (SEM).

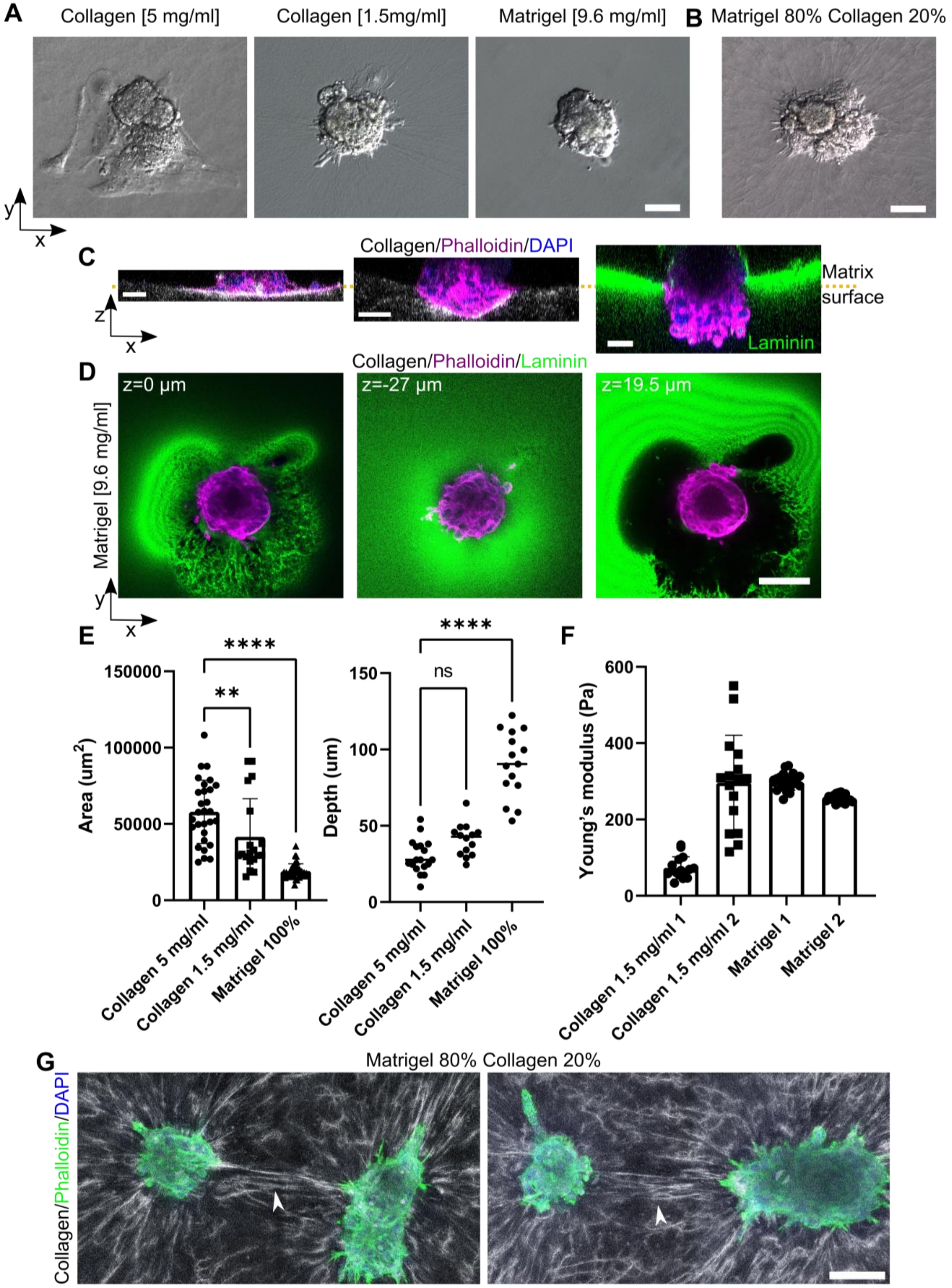

Fig. S3. Embryo implantation on 2D platform with varying matrix stiffness and composition. A) Snapshots of mouse embryos on 2D platform with varying matrix concentration and composition. The outgrowth size varies from substrate to substrate. Scale bar 100 μm. **B)** Snapshot of a mouse embryo

invading on a 2D platform formed with 80% Matrigel and 20% collagen. Scale bar 100 µm. **C)** Side-views of immunostained embryos with Phalloidin in red, DAPI in blue and laminin in green invading 2D platforms of different matrix concentration and composition. The collagen surface is indented. Scale bar 50 µm. **D)** Top-view projections of mouse embryo implanting on 2D platform formed with Matrigel. Laminin structure varies in function of distance to the embryo. Scale bar 100 µm. **E)** Area and depth of mouse embryo on collagen with different concentration (5 mg/ml and 1.5 mg/ml) and Matrigel (9.6 mg/ml). Area 0.60± 0.32, 0.41± 0.22 and 0.19±0.05mm^2 (n_{5mg}=39, $n_{1.5mg}$=26, $n_{Matrigel}$=39; N=2). Depth 29.7±11.0, 40.9±10.5 and 90.2±21.7um. n_{5mg}=18, $n_{1.5mg}$=14, $n_{Matrigel}$=15, N=2. **F)** Distribution of elastic modulus obtained by atomic force microscopy (AFM) for 2 samples on the 2D platform. Collagen of 1.5 mg/ml (both samples) has an elastic modulus of 188±144 Pa (n=31) and Matrigel 279±28 Pa (n=41). Error as standard deviation. **G)** Snapshot of mouse embryos aligning collagen (white arrows) on 80%Matrigel/20%collagen 2D platform stained for DAPI (blue) and Phalloidin (green). Scale bar 100 µm.

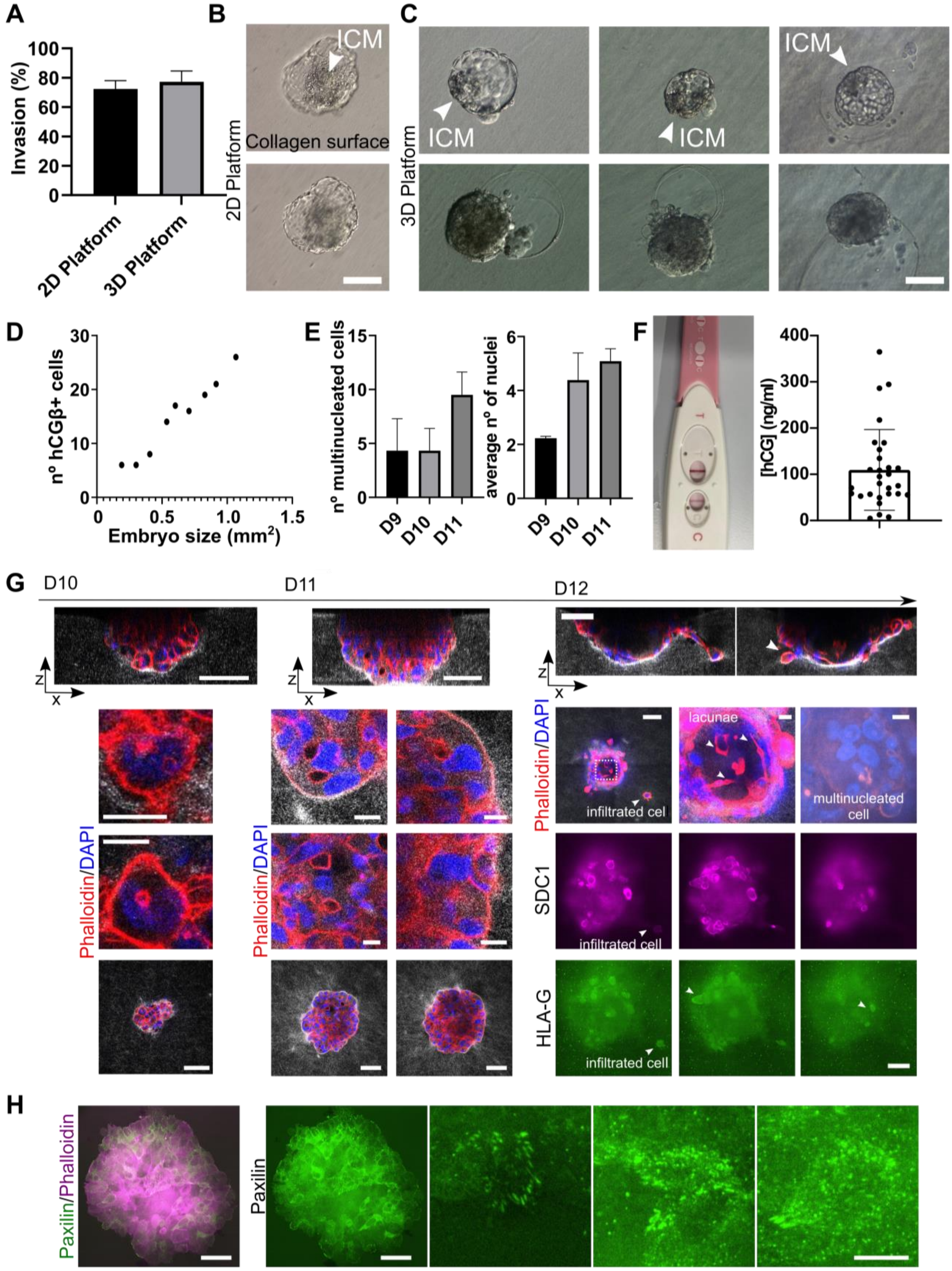

Fig. S4. Human embryo implantation. A) Graph showing adhesion rate on 2D platform and invasion rate on 3D platform. N_{2D}=66 embryos, N=16 and n_{3D}=38 embryos N=9. **B)** Snapshot of a human embryo

adhered to the 2D platform with the polar trophectoderm. Inner cell mass (ICM) indicated with arrow. **C)** Snapshots of human embryos in 3D platform at D6 and D8. The embryos have left a void and have translocated into the collagen matrix on the polar TE side. **D)** Area vs number of hCGβ positive cells in D9 human embryos. N=3 n=9 embryos **E)** Number of multinucleated cells per embryo and average number of nuclei in multinucleated cells at D9 (n=7), D10 (n=9) and D11 (n=2). **F)** Pregnancy test strip detecting human chorionic gonadotropin (hCGβ) secretion into the medium. Distribution of hCG concentration quantified by ELISA of implanting embryos after 2 days of culture in IVC1. Average concentration is 109.5±87.35 ng/ml n= 31 embryos N=2, error sd. **G)** Immunostaining of human embryos invading 2D matrix at D10, D11 and D12 phalloidin (red), DAPI (blue) HLA-G (green) and Syndecan (purple). Multinucleated cells have appeared by D10 with nascent lacunaes. Multinucleated cells and lacunaes are more prominent at D11 and at D12, large multinucleated cells have formed at the basal side. Cells at the basal side are positive for syndecan and HLA-G. Cell infiltration into the matrix were observed at D12. Representative embryo of D10=9, D11=2 and D12=1. Scale bar=100 µm. **H)** Immunostaining of a human embryo adhered to 2D ibidi plate at D9 with paxillin (green) and phalloidin (purple). The embryo forms prominent stress fibers and focal adhesions. Representative embryo of 4. All scale bars 100 µm insets 20 µm.

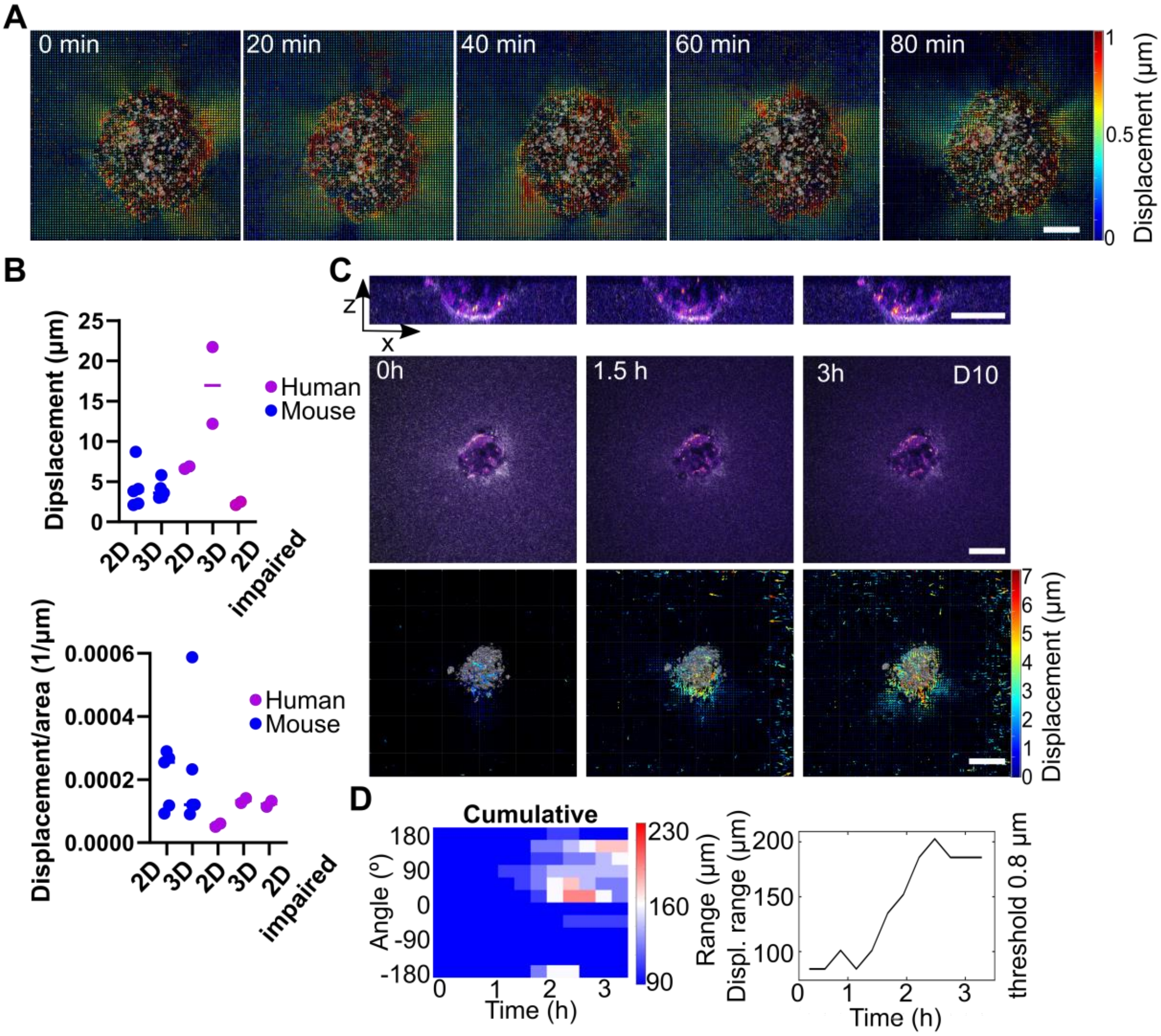

Fig. S5. Mechanics of human embryo implantation. A) Map of relative collagen displacement between two consecutive time points generated by single embryos on the 2D platform. The sites of traction fluctuate over time. The displacement amplitude is color-coded and the direction is indicated with arrows. **B)** Graphs showing collagen displacement after 3.5h in mouse (blue) and human embryos (purple) in 2D and 3D platforms. (bottom) Displacement normalized by embryo surface area assuming embryos are spheres. On the 2D platform the immersion of the sphere was calculated for the human embryo and the outgrowth area for mouse embryos. n_{mouse}=5 N_{mouse}=2, N_{human}=n_{human}=2 respectively. **C)** (top) Time-lapse images side and top view (single z plane) of a human embryo implanting in the 2D or 3D platform. The image of the matrix fibers and deformations were captured using light scattering. The embryo image was captured using autofluorescence and multiphoton illumination; (bottom) Corresponding maps of cumulative collagen displacement of the entire 3D volume generated by single embryos. The displacement amplitude is color-coded and the displacement direction is indicated with arrows. **D)** Graph showing the distance from the embryo at which a cut-off of 0.8 μm displacement is observed. The collagen displacement range increases over time around the embryo. (bottom) Heatmap of the radial distribution of cumulative displacement for a representative embryo on the 2D platform. Few displacement is observed compared to the invasion competent embryos. All scale bars=100 μm.

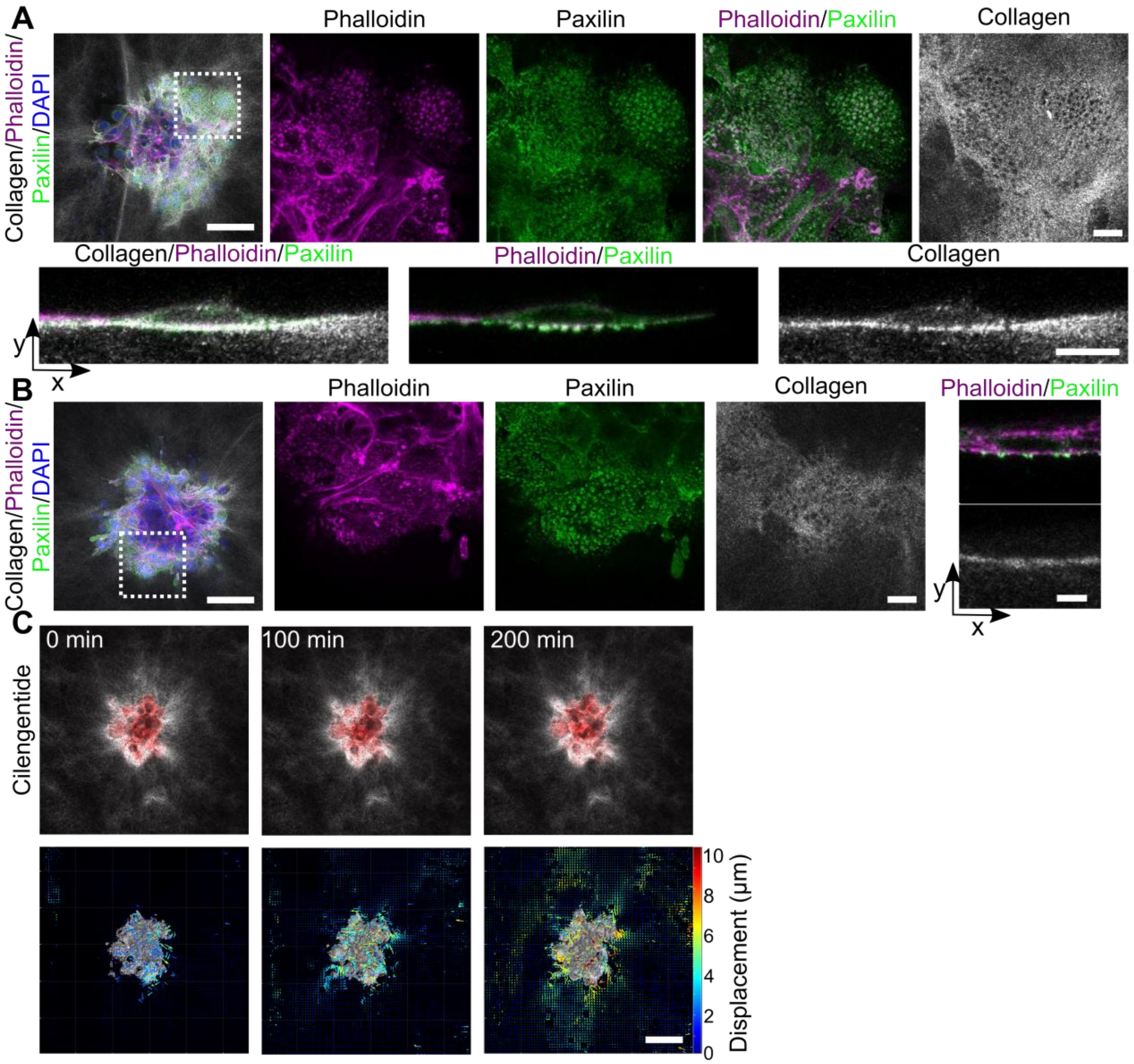

Fig S6. Focal adhesions and podosome-like structures A) and **B)** Snapshot of mouse embryo implanting on 2D platform stained for Phalloidin, Paxilin and DAPI. Ring structures can be seen in paxillin. Round holes in the collagen matrix are associated with the paxillin structures. **C)** Single z-plane time-lapse images of mouse embryos treated with 50µM Cilengentide expressing membrane-bound tdTomato (red). The matrix fibers and deformations were captured using light scattering. (bottom) Corresponding maps of cumulative collagen displacement of the entire 3D volume. The displacement amplitude is color-coded and the displacement direction is indicated with arrows. Scale bars 100 µm Insets and orthogonal view 20 µm.

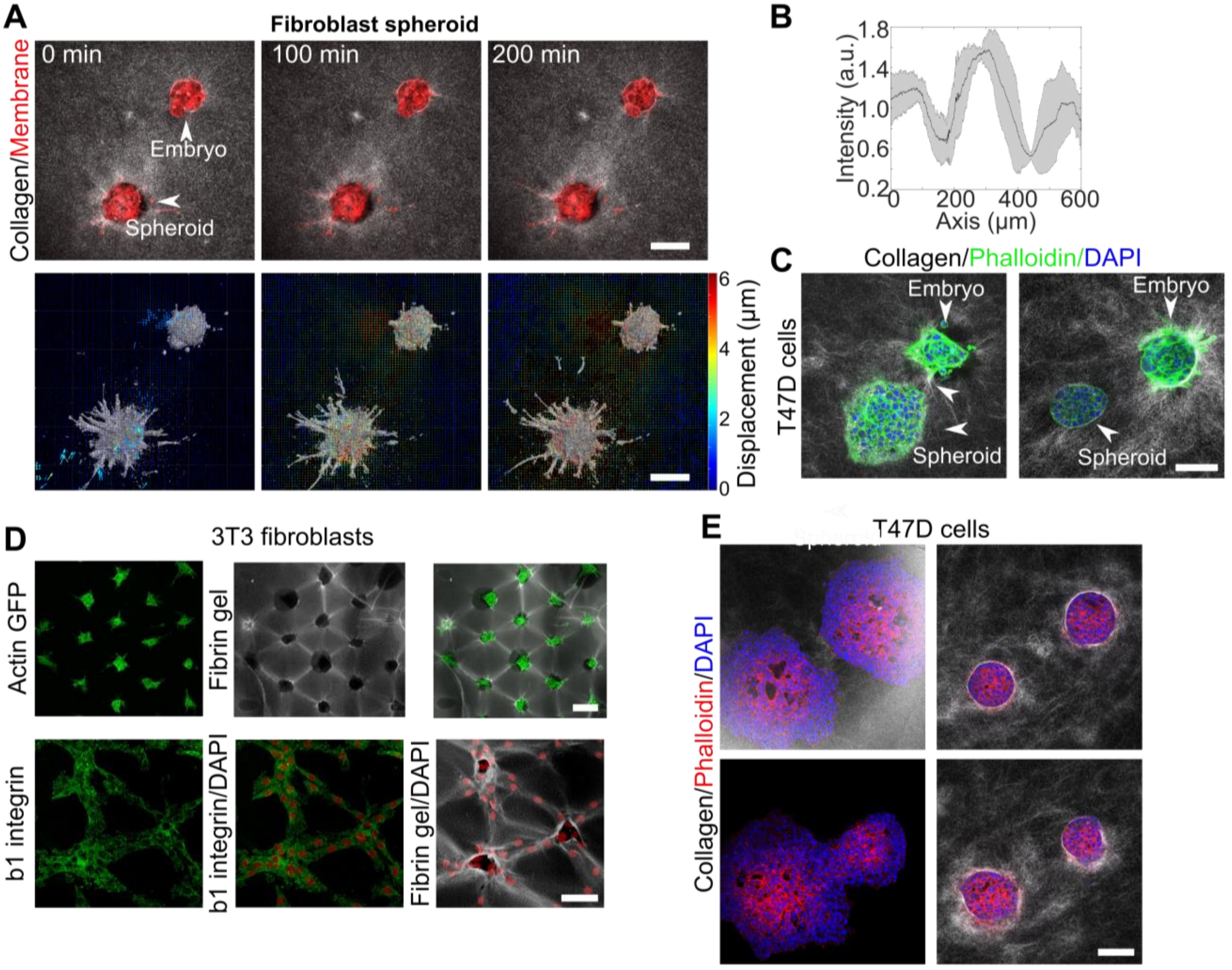

Fig. S7. Mechanical interaction in spheroid-embryo pairs A) (top) Time-lapse images of embryos expressing membrane-bound tdTomato and 3T3 fibroblast spheroid expressing Ephrin B2 (EPHB2) mRuby deforming the collagen matrix captured with light scattering. An increase in collagen intensity can be observed between the pair (mechanical bridge). (bottom) Map of cumulative collagen displacement applied by the embryo-3T3 spheroid pair. An increased collagen displacement can be observed along the connecting axis of the embryo and the spheroid. The displacement amplitude is color-coded and the displacement direction is indicated with arrows. **B)** Average intensity plot along the connecting axis of embryo-spheroid pairs. Spheroids are formed by 3T3 fibroblasts. The signal increases between the pair. n=5 pairs, N=2, the error is represented as SD. **C)** Snapshots of embryo spheroid pair. Spheroid is formed by T47D cells. Only very few alignment of collagen can be observed, mostly no mechanical interaction is visible. Representative images of 9 pairs. **D)** Snapshot of spheroid pairs formed by 3T3 fibroblasts stained for actin and b1 integrin. Spheroid form mechanical interaction. **E)** Snapshot of spheroid pairs formed by T47D cells stained for actin and DAPI. Cells show invasive behaviour on glass surface but no invasion of collagen or mechanical bridge formation was observed. Representative images of 7 pairs. All scale bars 100 µm.

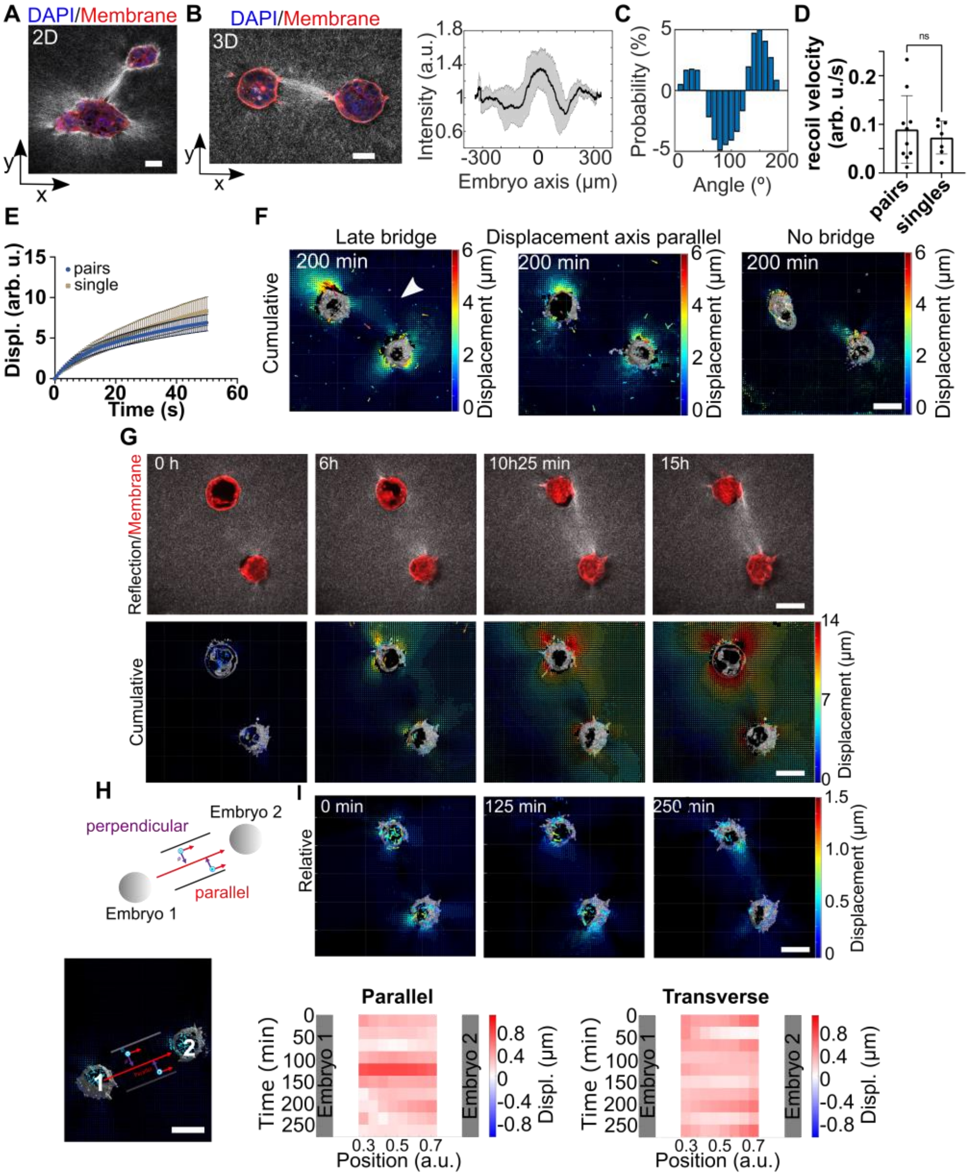

Fig. S8. Mechanical interaction with external force cues. A) Snapshot of an embryo pair implanted in the 2D platform expressing membrane-bound tdTomato and stained for DAPI. **B)** (left) Snapshot of a reflection image of two embryos implanting next to each other in the 3D platform. The collagen signal increase between the two embryos can be viewed in the image and the intensity plot on the right. N>3, n=11 pairs, the error is represented as the standard deviation. **C)** Distribution of collagen fibers along inter-embryo axis normalized by control region. More fibers are oriented along the connecting axis (0 and 180º) than transverse (90º). n=4 pairs. **D)** Recoil velocity of the matrix fibers after laser ablation between embryo pairs or next to individual embryos. N=3, n_{pairs} =10 n_{single} =7. (p>0.05, t-test). **E)** Plot of the displacement over time after laser ablation. N=3, n_{pairs}= 24 embryos.n_{single}=7 embryos. **F)** Snapshots of the digital volume correlation (DVC) analysis of cumulative displacement showing (left) late bridge formation (centre) perpendicular and (right) low collagen displacement with no bridge formation. **G)** (top) Time-lapse images of 15h embryo implantation (Figure 6B). Embryos expressing membrane-bound tdTomato deforming the collagen matrix captured with light scattering. (bottom) Map of cumulative collagen displacement generated by a pair of embryos. Increased displacement can be observed along the inter-embryo axis. The displacement amplitude is color-coded and the displacement direction is indicated with arrows. **H)** Schematic description of movement parallel and perpendicular to inter-embryo axis. **I)** (top) Map of relative collagen displacement between two time points generated by an embryo pair. The main displacement axis varies over time. (bottom left) Heatmap of the relative (bottom right) and perpendicular

collagen displacement parallel to the axis connecting the embryos. Displacement amplitude varies over time. Displacement is positive means the collagen becomes denser. All scale bar=100 μm.

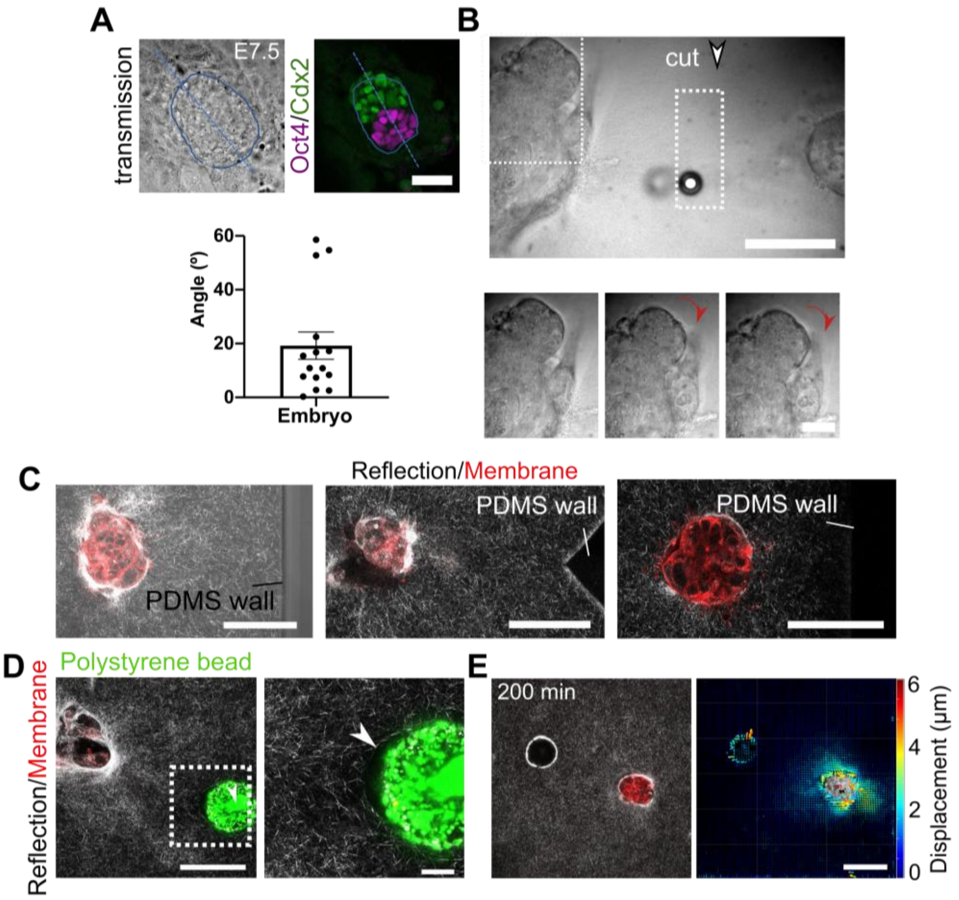

Fig. S9. Response to external mechanical force cues in 3D platform. A) (top) Brightfield and immunostaining image of Oct4 (Epiblast) and Cdx2 (Extraembryonic Ectoderm (ExE)) of a mouse embryo implanted in the 3D platform. The epiblast and ExE are outlined, and the axis indicated with a blue dashed line. Scale bar=50 µm. (bottom) Barplot of the angle variation of the outlined epiblast and ExE in transmission and with immunostaining. The average difference is 19.3 ± 5.1°. The error is represented as the SEM, n_{pairs} =14, N>3. The axis of the images obtained with transmitted light corresponds to the proximal-distal or embryonic-aembryonic axis. **B)** Time-lapse images of laser ablation. Dissection is followed by recoil, and embryo movement is highlighted in inset. Scale bar=50 µm and 20 µm. **C)** Snapshots of an implanting embryos expressing membrane-bound tdTomato implanting next to a polydimethylsiloxane (PDMS) wall in collagen imaged with light scattering. No apparent mechanical bridge is visible. **D)** Snapshot of implanting embryo expressing membrane-bound tdTomato and a green fluorescent polystyrene bead in collagen imaged with light scattering. No mechanical bridge is visible, but the collagen is detached from the bead on the side facing the embryo (inset and white arrow). **E)** Snapshot of embryos expressing membrane-bound tdTomato with a magnetic bead in vicinity, captured with light scattering and corresponding map of cumulative collagen displacement. No increased collagen displacement can be observed along the connecting axis between the embryo and the bead. The displacement amplitude is color-coded and the displacement direction is indicated with arrows. All scale bars=100 µm, inset 20 µm.

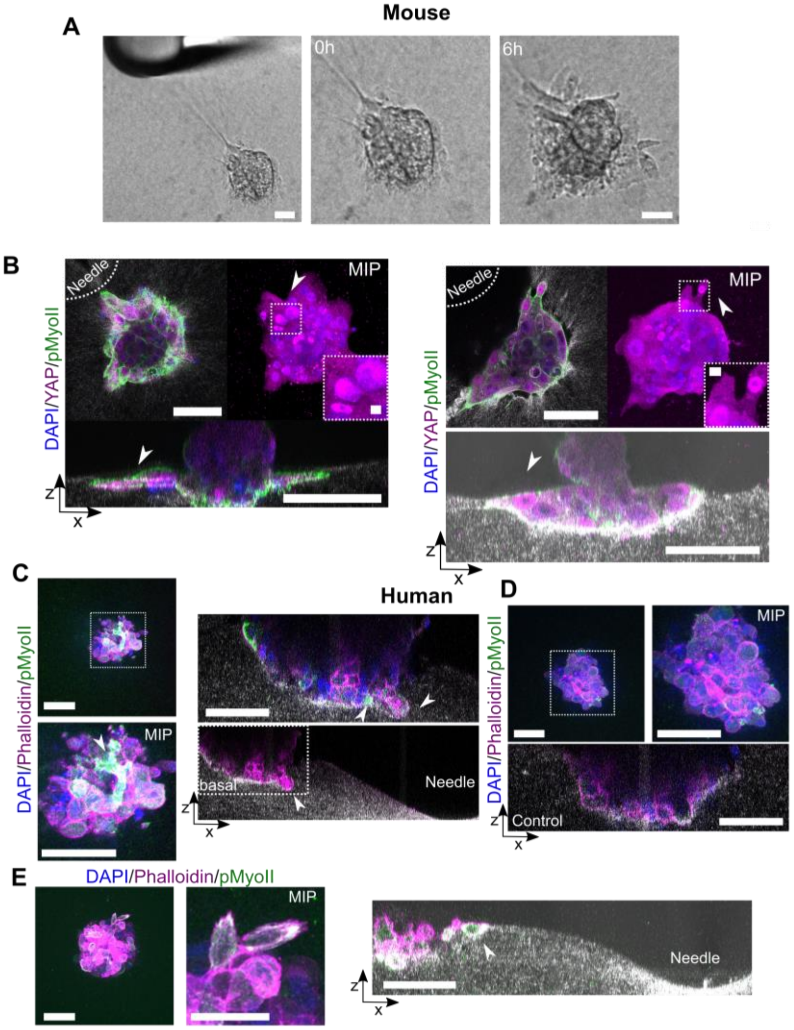

Fig. S10. Response to external mechanical force cues in 2D platform. A) Snapshots of an embryo before and after microneedle pressure. Scale bar=50 µm. **B)** Side view and maximal intensity projection (MIP) of confocal images of outgrowth immunostained for DAPI, YAP and phosphorylated myosin 2 (pMyoII) in implanted mouse embryos exposed to external force. Nuclear YAP localization is increased towards the microneedle localization (white arrows). Scale bar=100 µm, inset 10 µm. **C)** and **D)** Side view and maximal intensity projection (MIP) of implanted human embryos at D10 exposed to external force or control immunostained for DAPI, phosphorylated myosin 2 and phalloidin. The embryo forms cell projection towards the needle and accumulates myosin 2 at basal side (white arrows) when exposed to external force. Scale bar=100 µm. **E)** Side view and maximal intensity projection (MIP) of implanted human embryos at D10 exposed to external force immunostained for DAPI, phosphorylated myosin 2 and phalloidin. Cells on the collagen surface grow towards the needle and are enriched in phospho-myosin II. Scale bar 100 µm inset 50 µm.

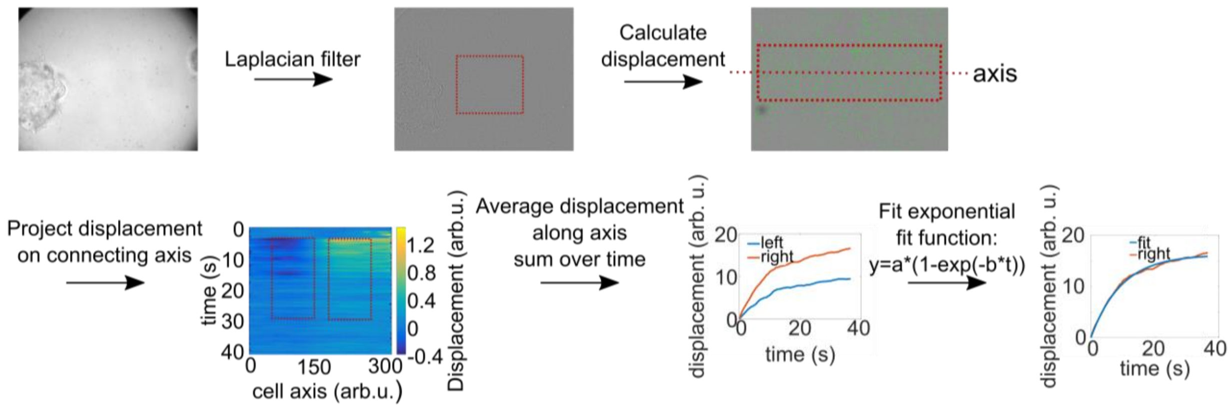

Fig. S11. Quantitative analysis of laserablation experiment. A Laplacian filter is applied to the raw data, then the displacement is determined via Kanade-lucas-tomasi (klt) tracker method on the area close to the cut. The displacement is projected on the axis to make it one dimensional and plotted in a heatmap over time. The moment of the cut shows the highest displacement. The displacement is averaged (red squares) and summed over time to generate 1 dimensional displacement curves. Then the exponential function $y=a*(1-\exp(-b*t))$ is fitted to the data.

Movie S1
Left panel: Time-lapse movie (single z-stack) of mouse embryo expressing membrane-bound tdTomato implanting on the 2D platform. Collagen images were acquired in reflection mode. Time in hh:mm. Right panel: digital volume correlation (DVC) analysis illustrating displacement of collagen matrix. Amplitude of displacement is colour coded, arrows indicate the direction of movement.

Movie S2
Left panel: Time-lapse movie (single z-stack) of mouse embryo expressing membrane-bound tdTomato implanting on the 3D platform. The collagen images were acquired in reflection mode. Time in hh:mm. Right panel: DVC analysis illustrating displacement of collagen matrix. Amplitude of displacement is colour coded, arrows indicate the direction of movement.

Movie S3
Left panel: Time-lapse movie (single z-stack) of a human embryo implanting on the 2D platform. The collagen images were acquired in reflection mode. Time in hh:mm. Center panel: z-section of a human embryo implanting on the 2D platform. Right panel: DVC analysis illustrating the displacement of the collagen matrix. The amplitude of the displacement is colour coded, arrows indicate the direction of movement.

Movie S4
Left panel: Time-lapse movie (maximum intensity projection) of a human embryo implanting in the 3D platform. The collagen images were acquired in reflection mode. The embryo was imaged with a multi-photon laser to excite the autofluorescent signal. Time in hh:mm. Right panel: DVC analysis illustrating the displacement of the collagen matrix. The amplitude of the displacement is colour coded, arrows indicate the direction of movement.

Movie S5
Left panel: Time-lapse movie (single z-stack) of a mouse embryo expressing membrane-bound tdTomato implanting on the 2D platform treated with 10 μM Dasatinhib. The collagen images were acquired in reflection mode. Time in hh:mm. The drug-treated embryo forms a limited outgrowth. Right panel: DVC analysis illustrating the displacement of collagen matrix. The amplitude of the displacement is colour coded, arrows indicate the direction of movement.

Movie S6
Left panel: Time-lapse movie (single z-stack) of of a mouse embryo and a spheroid formed by endometrial stromal cells at a distance of 200 μm expressing membrane-bound tdTomato and implanting on the 3D platform. The collagen intensity increases between the pair. Time in hh:mm. Right panel: DVC analysis illustrating the displacement of collagen matrix. Amplitude of displacement is colour coded, arrows indicate the direction of movement.

Movie S7
Left panel: Time-lapse movie (single z-stack) of two mouse embryos separated 200 μm expressing membrane-bound tdTomato and implanting on the 3D platform. The collagen images were acquired in reflection mode. The collagen intensity increases along the inter-embryo axis. Time in hh:mm. Right panel: DVC analysis illustrating the displacement of the collagen matrix. The amplitude of displacement is colour coded, arrows indicate the direction of movement.

Movie S8
Left panel: Time-lapse movie (single z-stack) of two mouse embryos at a distance of 200 μm expressing membrane-bound tdTomato implanting on the 2D platform. The collagen images were acquired in reflection mode. Time in hh:mm. Collagen intensity increases between two embryos. Right panel: DVC analysis illustrating the displacement of the collagen matrix. The amplitude of displacement is colour-coded, arrows indicate the direction of movement.

Movie S9

Time-lapse movie of bright field images during laser ablation of collagen at the intercept of two mouse embryos implanted in the 3D platform. The laser cut is followed by fast recoil and pulling of embryos. Time in mm:ss.

Movie S10
Time-lapse movie of bright field images during laser ablation of Matrigel at the intercept of two mouse embryos implanted in the 3D platform. No recoil or pulling of the embryos can be detected. Time in mm:ss.

Movie S11
Time-lapse movie (single z-stack) acquired on confocal microscope of two human embryos at a distance of 200 μm implanting in the 3D platform. Top panel: Brightfield signal of the human embryo pair. Bottom panel: The reflection captures the collagen intensity building up between the two embryos. Time in hh:mm.

Movie S12
Time-lapse movie of a mouse embryo implanting in the 2D platform. Pressure is applied by a micromanipulator next to the implanting embryo. The images were taken using transmitted light on a confocal microscope. Time in hh:mm.

Movie S13
Acquisition of a z-stack of a time-lapse movie of a mouse embryo implanting in the 2D platform. Pressure is applied by a micromanipulator next to the implanting embryo by keeping the needle still while moving the stage up and down for every time acquisition point. The images were taken using transmitted light on a confocal microscope. Time in hh:mm.

Movie S14
Time-lapse movie of human embryo implanting in the 2D platform. Pressure is applied by a micromanipulator next to the implanting embryo. The images were taken using transmitted light on a confocal microscope Time in hh:mm.
